# Supplementary material for: Study protocol: azithromycin therapy for chronic lung disease of prematurity (AZTEC) - a randomised, placebo-controlled trial of azithromycin for the prevention of chronic lung disease of prematurity in preterm infants
Source: BMJ Open. 2020 Oct 6;10(10):e041528. doi: 10.1136/bmjopen-2020-041528 (PMC7539578; doi:10.1136/bmjopen-2020-041528)
Supplement: Supplementary data [file bmjopen-2020-041528supp001.pdf]

Centre for Trials Research / Canolfan Ymchwil Treialon — University of Liverpool — The Newcastle upon Tyne Hospitals NHS Foundation Trust — UCL — Funded by NIHR National Institute for Health Research

<<NHS Organisation Logo>>
<Trust/Site address 1>
<Trust/Site address 2>
<Trust/Site address 3>
<postcode>

AZTEC

Azithromycin Therapy for Chronic Lung Disease

Parent Information Sheet

Summary

This is a brief summary of a research study called **AZTEC**. We would like to invite your baby to join our study because your baby may be, or has been, born early.

Babies born early are at risk of developing lung problems. Giving an extra medicine called azithromycin, (an 'antibiotic'), to a baby born early, may be able help their lung problems. However, at the moment, we do not know whether babies who have this medicine will benefit compared to babies who do not receive the medicine. Our hospital is one of many helping in a UK-wide study to find out whether the medicine should be used routinely in the future for premature babies. We would like approximately 800 families to take part, in total.

If, after reading this information and talking with others, you wish for your baby to take part, we will ask you to sign a consent form and your baby will then be entered into the study. We will collect information about your baby's progress in hospital and would like to follow them up at one and two years of age.

Taking part in the study is entirely up to you. If you decide not to take part, this will not affect the care your baby receives. You do not have to give a reason if you do not want to take part, although providing your reasons may be helpful to us if you are willing. The rest of this leaflet explains the study in more detail and describes what being in the study would mean for you and your baby.

Thank you for taking the time to read this leaflet.

We would like to invite your baby to take part in our study called AZTEC

We understand and appreciate that this is a very difficult time for you and your family. However, we think it is important that you know about a study that this hospital is taking part in for babies born early.

We would like to ask your permission for your baby to take part in this study. Before you decide, it is important for you to understand why the research is being done and what is involved.

What is the AZTEC Study about?

Babies born early (under 30 weeks) are at risk of developing lung problems after birth. A major reason for this is that the lungs are not fully developed. This often means that after birth, babies born early may need some extra medicines and sometimes help with their breathing. Babies born early often develop inflammation (soreness and redness) of their lungs, sometimes because of germs (including one called *Ureaplasma)*.

Azithromycin is a medicine which can reduce inflammation and treats infection in the lungs. Reducing either, or both of these might help the lung problems of babies born early. The best way to find out is to compare babies who are given azithromycin with those who are not.

Why has my baby been asked to take part?

We are inviting your baby to take part in this study because they are a patient at one of the hospitals taking part and have been born under 30 weeks of pregnancy and need some help with their breathing.

Does my baby have to take part?

No, taking part is voluntary. It is up to you to decide whether or not you want your baby to take part. If you choose to let your baby take part you can also choose to stop at any time without giving a reason, although providing your reasons may be helpful to us if you are

willing. The standard of care your baby will receive now or in the future will be the same whether they take part or not.

What will my baby and I have to do if we take part?

A member of the clinical team will talk to you first in more detail and you will be able to ask any questions that you have. If you have had all of your questions answered and are happy for your baby to take part then you will be asked to sign a consent form to confirm you would like your baby to participate. You will be given a copy of the consent form and the information sheet to keep.

In research studies, we often test the medicine against a dummy medicine (also called a placebo) to see if the medicine works. A computer program will decide if your baby receives the azithromycin once a day for ten days or the dummy medicine, (water), once a day for ten days. Neither you, nor the team looking after your baby will know which medicine the baby is on. However, if the team looking after your baby need to know what your baby is receiving then they can find out quickly. The germs may be acquired before birth or at delivery, so it is best to start the treatment as early as possible and within 72 hours of birth.

We will also collect fluid samples from your baby's breathing tubes or from their nose. This is done regularly for all babies who need help with their breathing, but the fluid is not normally kept. The fluid will be tested for germs and inflammation. Samples of your baby's stool (poo) will also be taken to look if azithromycin has an effect on normal bacteria. All of these will only be done if your baby is well enough. All other aspects of your baby's care will be exactly the same as if they were not in the study.

We shall collect details of your baby until 36 weeks of gestation or until the baby leaves the hospital if this is sooner. Some babies need oxygen for a variable length of time to help with their breathing - if your baby needs oxygen at 36 weeks' gestation then we shall carry out a short test to find out how much oxygen they require.

To find out if the treatment improves breathing when your baby grows up, we would like to contact you and your GP when your baby is one and two years old to see how they are doing. We will ask you to fill in questionnaires about their breathing and general health. We would also like to use routinely collected health data (information hospitals collect when looking after your baby), which is held by this hospital, NHS Digital, and from the National Neonatal Research database, which collects data on the follow-up of premature babies and children. Information such as your baby's name, NHS/CHI number will be used for this purpose, but the data viewed by Cardiff University researchers will not be identifiable. All information will be stored securely.

Timing of treatment and sample collection

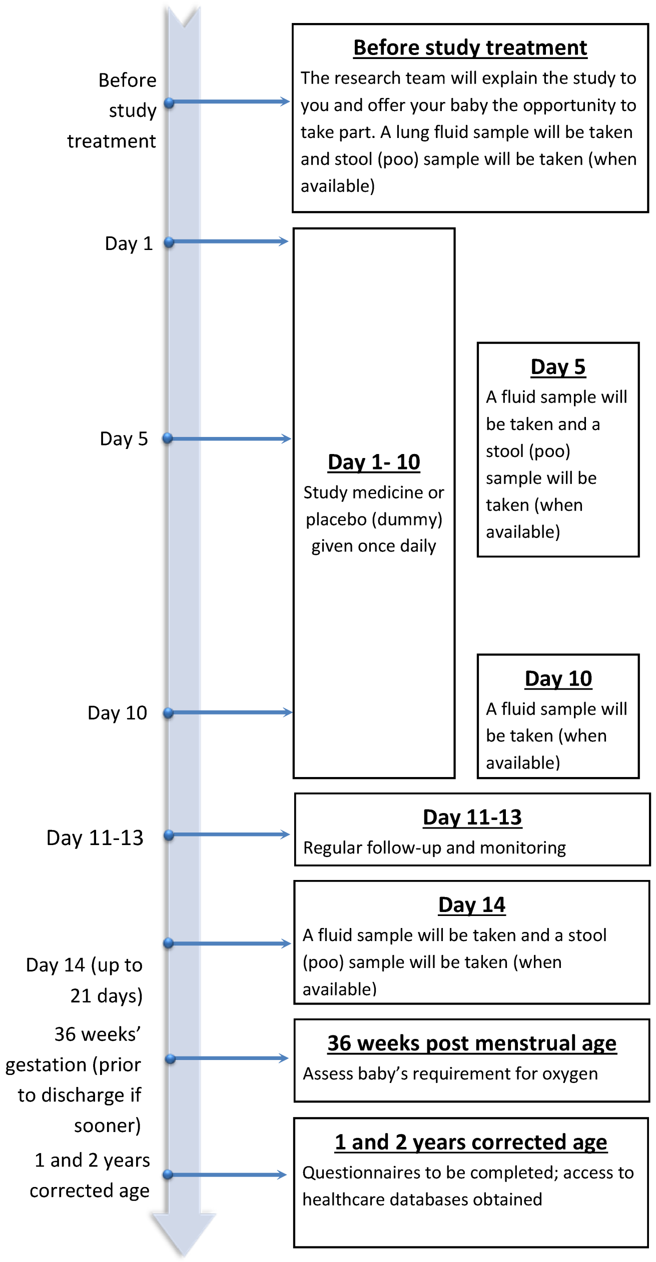

What are the benefits and risks of my baby taking part?

Azithromycin has been used for a long time in children and has been used in other research studies in premature babies. Like all medicines, antibiotics can sometimes cause side-effects; these are uncommon, but we know that some babies may develop some soreness of the tummy or slightly looser stools. In older patients, azithromycin may affect the rhythm of the heart. There is no evidence that this happens in babies, but it is something we will monitor closely.

Although there are no certain benefits to you or your baby in taking part in the study, we hope the information we get might improve treatment and health outcomes for other babies in the future.

Will my baby's participation be kept confidential?

Yes. All the information collected about your baby during the study will be handled according to all applicable ethical and legal requirements.

Your baby's personal information will be kept strictly confidential and will only be accessed by people working on the study or working to ensure the study is being run correctly.

Your baby will be allocated a study number, which will be used to identify them on each paper form. Your full name, your baby's full name, and your baby's NHS number will be included on their consent form and a copy of this will be sent to the coordinating centre for the study, the Centre for Trials Research (CTR), based at Cardiff University. Every effort will be made to ensure that any information about your baby that leaves the hospital will have your baby's identifiers removed so that your baby cannot be recognised from it; this information will usually be removed by a member of the study team at your baby's hospital but may also be removed by the CTR upon receipt.

What will happen to the samples from my baby?

Any samples taken will be stored securely until they are transferred to Cardiff University. We shall test the samples in our laboratories at Cardiff, but some tests may be done by other university laboratories or commercial companies who have the appropriate expertise. In all cases, samples will be given a special code so that the person doing the laboratory tests will not know which baby the sample came from. We will record results on a special database where all the information is linked to this code. We shall use the baby's nose, lung and stool samples to look for DNA from germs. When we extract the DNA from the samples, DNA from both the baby and germs is unavoidably extracted. For this study we shall not use the baby's DNA. However, with your permission any remaining samples, including nose and lung fluid, stool, and DNA (baby and bacterial), may be stored for future research into chronic lung disease of prematurity.

The samples will be anonymised before use in future studies and may be accessed by researchers in the UK and abroad. The research may include genetic (e.g. DNA) or commercial research. You may withdraw your consent for the storage and future use of your baby's samples at any point by contacting the Centre for Trials Research (www.cardiff.ac.uk/centre-for-trials-research).

If you do withdraw your consent your baby's samples will not be used in any subsequent studies and will be destroyed according to locally approved practices. Any samples already distributed for use in research prior to the withdrawal of consent will continue to be used in that study and any samples remaining at the end of the study will be destroyed.

What happens if I change my mind about my baby taking part?

If at any point you decide you want your baby to stop taking part in the study, the care your baby receives will not be affected. If you do decide for your baby to stop taking part we would stop giving study treatment (if not finished) and ask you if you would like your baby to:

- continue to complete study follow up (data collection and whether stool/fluid samples can be taken)
- continue to be followed up at 1 and at 2 years of age, or
- stop taking part with no more study follow up.

If you withdraw your baby from the study, we will keep the information about your baby that we have already obtained. We may be required to collect some limited information about your baby and about side effects your baby may have as a result of taking part in the trial. This will only be collected if required by the Regulatory Authorities.

What will happen to the results of the study?

We aim to publish the results in medical journals. Confidentiality will be ensured at all times and you or your baby will not be identified in any publications. A summary will be published on the study website.

What if there is a problem?

If you have a concern about any aspect of this study, you should ask to speak with one of your baby's research team who will do their best to answer your questions. If you remain unhappy and wish to complain formally, you can do this through the NHS Complaints Procedure. Details can be obtained from the hospital.

If in the unlikely event your baby has been harmed by taking part in this study, you may have grounds for legal action and could seek compensation through the sponsor, who has appropriate insurance-related arrangements in place. If your baby is harmed and it is due to any routine clinical treatment or negligence, then the <<hospital institution>> indemnity arrangements will apply. If you wish to complain about any aspect of the way you or your baby has been treated, you may use the normal <<hospital institution>> complaints procedures; <<hospital advice service>> at your hospital will advise you about this (their contact details are at the end of this leaflet).

Additional information

Cardiff University is the sponsor and the data controller for this study. This means that they are responsible for looking after your baby's information and using it properly. They will be using information from your baby's medical records in order to undertake this study. The CTR at Cardiff University will keep identifiable information about your baby for 25 years after the study has finished. Arrangements for confidential destruction will then be made.

Your rights to access, change or move your baby's information are limited, as your baby's information needs to be managed in specific ways in order for the research to be reliable and accurate. If you withdraw your baby from the study, Cardiff University will keep the information about your baby that we have already obtained. To safeguard you and your baby's rights, they will use the minimum personally-identifiable information possible.

This hospital will use your baby's name, NHS/CHI number and contact details to make sure that relevant information about the study is recorded for your baby's care, and to oversee the quality of the study. This hospital will pass these details to Cardiff University so individuals from Cardiff University, and regulatory organisations may look at your baby's medical and research records to check the accuracy of the research study. The only other people at Cardiff who will have access to information that identifies your baby will be people who need to contact you for study follow-up or to audit the data collection process. This hospital will keep identifiable information about you from this study for 25 years after the study has finished.

AZTEC is funded by National Institute of Health Research, HTA Programme (HTA Project: 16/111/106). The study is registered on a public database of clinical trials called ISRCTN (ISRCTN11650227). For further details of national databases reviewed for this study please refer to the website <<URL>>. You can also find out more about how your information will be used on these webpages.

The research has been approved by Wales Research Ethics Committee 2, NHS Heath Research Authority, and by the Medicines and Healthcare Products Regulatory Agency (MHRA) who have agreed this study is being conducted in an appropriate manner.

Contacts for further information

If you would like more information or have any questions about the AZTEC study, please talk to:

Principal Investigator: **<PI NAME>**

Research Nurse: **<RN NAME>**

Telephone: **<number>**

Or visit the website: <<URL>>

If you wish to discuss the study with someone independent of the research team you can contact the local NHS Patient Advice and Liaison Service (PALS) on: <telephone number>

Thank you taking the time to read this information sheet

AZTEC
Azithromycin Therapy for Chronic Lung Disease

<<NHS Organisation Logo>>
<Trust/Site address 1>
<Trust/Site address 2>
<Trust/Site address 3>
<postcode>

Consent Form

Study ID: | A | Z | | | | | ***NHS/CHI NUMBER:*** | | | | | | | | | | |

To be completed by the Parent/Guardian

Once you have read and understood each statement please enter your initials in each box	Initial
1. I have read and understood the information sheet for the AZTEC trial (Version 5.0, dated 20/06/2019). I have had the opportunity to ask questions and have had these answered satisfactorily.	
2. I understand that my baby's participation is voluntary and that I am free to withdraw my baby from the study at any time, without giving a reason, and without my baby's care or legal rights being affected. I understand that in some cases further information about any unwanted effects of my baby's treatment may need to be collected by the study team.	
3. I understand that my baby's data will be retained for a maximum of 25 years and that they will be stored in a confidential manner.	
4. I understand that information about both mother and baby that is held and maintained by NHS Digital and other central UK NHS bodies, may be collected from medical records and other health-related records. These may be looked at by the research team and responsible practitioners during the trial. The sponsor, NHS Organisation and Regulatory Authorities may also look at the data collected during the study. I give permission for these individuals to have access to these records and for them to be used in this research on the understanding that all information will remain confidential.	
5. I understand that samples of my baby's lung fluid, stool and nasal fluid will be collected for this study. I understand that my baby's DNA will be extracted alongside the bacterial DNA, but will not be used in the AZTEC study.	
6. I agree to allow information or results arising from this study to be used in future healthcare and/or medical research in an anonymised form.	
7. I give permission for a copy of my baby's consent form to be sent to the CTR at Cardiff University (where it will be kept in a secure location), to allow confirmation that my consent was given.	
8. I agree for my baby to take part in the above study.	
Below are optional statements:	
9. I agree for my baby's follow up data on applicable databases (at this hospital, NHS digital/NHS Wales Informatics Service/ISD Scotland and the National Neonatal Research Database) to be reviewed by Cardiff University researchers.	
10. I agree for me and my GP to be contacted by Cardiff University for the purposes of follow up when my baby is one and two years corrected age. I understand that contact details will be kept strictly confidential (according to the 2018 General Data Protection Regulation) and that no personal information will be used in study reports or publications.	
11. I agree to gift any of my baby's remaining samples (including my baby's DNA) to be used in future chronic lung disease of prematurity research in the UK and abroad, which may include genetic (e.g. DNA) and commercial research. I understand I am free to withdraw my consent to future research at any point and that all samples will be destroyed as detailed in the information sheet	
12. I agree that I may be contacted in the future in relation to other research studies	

Baby's name (please print): ______________________________

Name of parent/guardian (please print): ______________________________ Your signature: __________________________

Relationship to baby: ____________________________ Date: ______________

2nd Parent name, (please print): __________________________ Your signature: __________________________ Date: ______________

Researcher name (please print): _____________________________ Signature: ______________________________ Date: ______________
