# Supplementary material for: Study protocol: azithromycin therapy for chronic lung disease of prematurity (AZTEC) - a randomised, placebo-controlled trial of azithromycin for the prevention of chronic lung disease of prematurity in preterm infants
Source: BMJ Open. 2020 Oct 6;10(10):e041528. doi: 10.1136/bmjopen-2020-041528 (PMC7539578; doi:10.1136/bmjopen-2020-041528)
Supplement: Author's manuscript [file bmjopen-2020-041528.draft_revisions.pdf]

BMJ Open

BMJ Open is committed to open peer review. As part of this commitment we make the peer review history of every article we publish publicly available.

When an article is published we post the peer reviewers' comments and the authors' responses online. We also post the versions of the paper that were used during peer review. These are the versions that the peer review comments apply to.

The versions of the paper that follow are the versions that were submitted during the peer review process. They are not the versions of record or the final published versions. They should not be cited or distributed as the published version of this manuscript.

BMJ Open is an open access journal and the full, final, typeset and author-corrected version of record of the manuscript is available on our site with no access controls, subscription charges or pay-per-view fees (http://bmjopen.bmj.com).

If you have any questions on BMJ Open's open peer review process please email info.bmjopen@bmj.com

BMJ Open

Azithromycin Therapy for Chronic Lung Disease of Prematurity (AZTEC): A randomised, placebo-controlled trial of azithromycin for the prevention of chronic lung disease of prematurity in preterm infants

Journal:	*BMJ Open*
Manuscript ID	bmjopen-2020-041528
Article Type:	Protocol
Date Submitted by the Author:	10-Jun-2020

Complete List of Authors:	Lowe, John; Centre for Trials Research, University of Cardiff<br>Gillespie, David; Centre for Trials Research, University of Cardiff ,<br>Hubbard, Marie; University Hospitals of Leicester NHS Trust<br>Zhang, Lei; Cardiff University School of Medicine,<br>Kirby, Nigel; Cardiff University, Centre for Trials Research<br>Pickles, Timothy; Cardiff University, South East Wales Trials Unit (SEWTU)<br>Thomas-Jones, Emma; Cardiff University, Centre for Trials Research<br>Turner, Mark; Liverpool Women's Hospital NHS Foundation Trust,<br>Klein, Nigel; University College London<br>Marchesi, Julian; Cardiff University, School of Biosciences; Imperial College London, Department of Metabolism, Digestion and Reproduction, Faculty of Medicine<br>Hood, Kerenza; Cardiff University,<br>Berrington, Janet; Newcastle Upon Tyne Hospitals NHS Foundation Trust<br>Kotecha, Sailesh; Cardiff University School of Medicine, Department of Child Health, Institute of Molecular & Experimental Medicine

Keywords:	NEONATOLOGY, Paediatric thoracic medicine < PAEDIATRICS, Chronic airways disease < THORACIC MEDICINE

SCHOLARONE™
Manuscripts

I, the Submitting Author has the right to grant and does grant on behalf of all authors of the Work (as defined in the below author licence), an exclusive licence and/or a non-exclusive licence for contributions from authors who are: i) UK Crown employees; ii) where BMJ has agreed a CC-BY licence shall apply, and/or iii) in accordance with the terms applicable for US Federal Government officers or employees acting as part of their official duties; on a worldwide, perpetual, irrevocable, royalty-free basis to BMJ Publishing Group Ltd ("BMJ") its licensees and where the relevant Journal is co-owned by BMJ to the co-owners of the Journal, to publish the Work in this journal and any other BMJ products and to exploit all rights, as set out in our licence.

The Submitting Author accepts and understands that any supply made under these terms is made by BMJ to the Submitting Author unless you are acting as an employee on behalf of your employer or a postgraduate student of an affiliated institution which is paying any applicable article publishing charge ("APC") for Open Access articles. Where the Submitting Author wishes to make the Work available on an Open Access basis (and intends to pay the relevant APC), the terms of reuse of such Open Access shall be governed by a Creative Commons licence – details of these licences and which Creative Commons licence will apply to this Work are set out in our licence referred to above.

Other than as permitted in any relevant BMJ Author's Self Archiving Policies, I confirm this Work has not been accepted for publication elsewhere, is not being considered for publication elsewhere and does not duplicate material already published. I confirm all authors consent to publication of this Work and authorise the granting of this licence.

For peer review only

Azithromycin Therapy for Chronic Lung Disease of Prematurity (AZTEC): A randomised, placebo-controlled trial of azithromycin for the prevention of chronic lung disease of prematurity in preterm infants

John Lowe[1], David Gillespie[1], Marie Hubbard[2], Lei Zhang[3], Nigel Kirby[1], Tim Pickles[1], Emma Thomas-Jones[1], Mark Turner[4], Nigel Klein[5], Julian Marchesi[6], Kerenza Hood[1], Janet Berrington[7], Sailesh Kotecha[3]

1 Centre for Trials Research, College of Biomedical and Life Sciences, Cardiff University, Cardiff, UK

2 University Hospitals of Leicester NHS Foundation Trust, Leicester, UK

3 Department of Child Health, School of Medicine, Cardiff University, Cardiff, UK

4 Women and Children's Health, University of Liverpool, UK

5 GOS Institute of Child Health, University College London, London, UK

6 School of Biosciences, Cardiff University, Cardiff, UK

7 The Newcastle upon Tyne Hospitals NHS Foundation Trust, Newcastle upon Tyne, UK

Corresponding Author: Professor Sailesh Kotecha
Department of Child Health
School of Medicine
Cardiff University
Heath Park
Cardiff
CF14 4XN
United Kingdom
Email: KotechaS@cardiff.ac.uk
Telephone: 029 20 74 4187
Fax: 029 20 74 4283

For peer review only - http://bmjopen.bmj.com/site/about/guidelines.xhtml

Abstract

Introduction

Chronic Lung Disease of Prematurity (CLD), also known as Bronchopulmonary dysplasia (BPD), is a cause of significant respiratory morbidity in childhood and beyond. Coupled with lung immaturity, infections (especially by *Ureaplasma* spp.) are implicated in the pathogenesis of CLD through promotion of pulmonary inflammation. Azithromycin, which is a highly effective against *Ureaplasma* spp. also has potent anti-inflammatory properties. Thus, azithromycin therapy may improve respiratory outcomes by targeting infective and inflammatory pathways. Previous trials using macrolides have not been sufficiently powered to definitively assess CLD rates. To address this, the Azithromycin Therapy for Chronic Lung Disease (AZTEC) trial aims to determine if a 10-day early course of intravenous azithromycin improves rates of survival without CLD when compared to placebo with an appropriately powered study.

Methods and Analysis

796 infants born at less than 30 weeks' gestational age who require at least 2 hours of continuous respiratory support within the first 72 hours following birth are being enrolled by neonatal units in the UK. They are being randomised to receive a double-blind, once daily dose of intravenous azithromycin (20mg/kg for 3 days, followed by 10mg/kg for a further 7 days), or placebo. CLD is being assessed at 36 weeks' postmenstrual age. Whether colonisation with *Ureaplasma* spp. prior to randomisation modifies the treatment effect of azithromycin compared to placebo will also be investigated. Secondary outcomes include necrotising enterocolitis, intraventricular/cerebral haemorrhage, retinopathy of prematurity and nosocomial infections), development of antibiotic resistance; adverse reactions will be monitored.

Ethics and dissemination:

Ethics permission has been granted by Wales Research Ethics Committee 2 (Ref 18/WA/0199), and regulatory permission by the Medicines and Healthcare Products Regulatory Agency (CTA reference 21323/0050/001-0001). The study is registered on ISRCTN (ISRCTN11650227). The study is overseen by an independent Data Monitoring Committee and an independent Trial Steering Committee.

For peer review only

For peer review only - http://bmjopen.bmj.com/site/about/guidelines.xhtml

Strengths and limitations of this study

- This is a randomised, double-blind placebo-controlled trial assessing an important outcome, and using an intervention which could be readily implemented in to clinical practice
- If the assumptions underlying the sample size calculation are valid, this study will be appropriately powered for the primary outcome
- The study will also help address the role of pulmonary *Ureaplasma* spp. colonisation in the development of CLD
- Universal administration of azithromycin may alter antibiotic resistance patterns, which are also being investigated in this study
- Further work will be required to investigate the mechanistic action of azithromycin including on the lung and gut microbiota

Introduction

Chronic Lung Disease of Prematurity (CLD), also known as Bronchopulmonary dysplasia (BPD), is a major cause of neonatal death in infants born prematurely. It is clear that survivors of CLD have adverse respiratory outcomes in childhood and beyond [1-3].

Despite the advances in neonatal care, rates of CLD have not markedly changed. This is largely because the survival of the most immature infants born at the limits viability (22 – 23 weeks' gestation) means that the proportion of preterm infants born at 28 weeks of less of gestation has increased over the last thirty years [4]. Consequently, the etiological definition of CLD was modified to represent the structural immaturity of the lungs which are in the cannalicular/saccular stages of development [5 6]. The pathophysiology is complex and results from continual lung injury caused by inflammatory processes coupled with repair/remodelling and resultant fibrosis [7]. In contrast, the historical picture of CLD was largely characterised by parenchymal tissue damage caused by volutrauma, barotrauma and by high levels of oxygen required to sustain life [8].

Infections, both antenatal (e.g. chorioamnionitis) and nosocomial contribute to a cytokine-mediated inflammatory cascade which peaks between 7-10 days of life [9 10]. *Ureaplasma* spp. (class Mollicutes) are the smallest free living organisms and have long been implicated in the pathogenesis of CLD [11]. These commensal bacteria form part of the normal vaginal bacteria microbiota thus are readily transmitted to the uterus via the cervix or acquired during vaginal delivery. Lacking a cell wall, they are readily transferred to the lung where they colonise the respiratory mucosa and directly, or, in

For peer review only - http://bmjopen.bmj.com/site/about/guidelines.xhtml

conjunction with aforementioned iatrogenic factors, indirectly promote inflammation largely through recruitment of neutrophils to the lung. Our recent systematic review and meta-analysis reported that *Ureaplasma* spp. colonisation was strongly associated with diagnosis of CLD at 36 weeks' postmenstrual age (odds ratio 2.22; 95% CI 1.42, 3.47) which was largely independent of gestational age when investigated with meta-regression [12].

Current treatments for the prevention of CLD are largely supportive and include optimising use of early non-invasive respiratory support and preventing infections. Use of systemic corticosteroids is considered in cases where the infant is unable to be weaned from invasive ventilation. However, it is well-established that early, high-dose use of corticosteroids is associated with adverse neurodevelopmental outcomes including cerebral palsy [13].

Due to risks associated with use of corticosteroids, other treatments, or ideally preventive strategies, with improved safety profiles are urgently required for CLD. An interest in macrolide antibiotics, and the potential for improving lung outcomes via eradication of *Ureaplasma* was established in the 1990s. However, early trials of erythromycin were underpowered and did not reduce the incidence or severity of CLD [14]. A study trialling clarithromycin treatment for 10 days reported a reduction in CLD (2.9% Vs. 36%) when compared to placebo but importantly only initiated delayed randomised therapy if the infant was culture-positive for *Ureaplasma* [15]. More contemporary data exists with azithromycin which is an important adjunct therapy for numerous respiratory conditions, including cystic fibrosis and chronic obstructive pulmonary disease (COPD). Consistent with other macrolides, azithromycin inhibits bacterial protein synthesis through blinding to the 50s bacterial ribosomal subunit [16]. Moreover, azithromycin is attractive as it also uniquely exhibits well-characterised immunomodulatory effects through suppression of Nuclear factor-kappaB, decreasing neutrophilic pulmonary inflammation by limiting the production of pro-inflammatory cytokines such as IL-6 and IL-8 [17]. Furthermore, since azithromycin is concentrated in leukocytes, it may be actively transported and released at the site of infection [18].

Several proof of concept and dose-finding studies have already been completed. The meta-analysis by Nair and colleagues showed that azithromycin treatment is associated with a 14% reduction in the combined outcome of CLD/death when compared to placebo [19]. Dose-finding work in respect of *Ureaplasma* spp. eradication has been undertaken in a series of studies by Viscardi and colleagues [20-22]. Importantly, all previous studies did not note any serious adverse reactions to azithromycin and add to the existing safety information [23]. Antimicrobial resistance amongst *Ureaplasma* remains low [24].

For peer review only - http://bmjopen.bmj.com/site/about/guidelines.xhtml

Given the Nair meta-analyses and lack of adequately powered studies, and given the reported association of presence of pulmonary inflammation and *Ureaplasma* with development of CLD, there is a compelling case for a definitive, adequately powered study to investigate the effectiveness of azithromycin on reducing rates of CLD in preterm infants. The Azithromycin Therapy for Chronic Lung Disease trial (AZTEC) is determining if a 10-day course of intravenous azithromycin improves rates of survival without CLD at 36 weeks' postmenstrual age when compared to placebo.

Methods and analysis

Primary Objective

The primary objective of the AZTEC trial is to assess the effectiveness of a 10-day course of azithromycin on improving survival without physiologically-defined CLD in infants born at <30 weeks' gestational age.

Design

AZTEC is a double-blind, randomised, placebo-controlled trial. The total samples size is 796 preterm-born infants; each are randomised individually (including infants from a multiple birth). Follow-up is undertaken at 36 weeks' post-menstrual age (PMA) to assess CLD status at 36 weeks' postmenstrual age, with final follow up a discharge from hospital.

Setting

Infants are being enrolled from UK tertiary neonatal units which are designated Level 3 (regional neonatal intensive care units), and followed up at their local hospital if transferred. Infants are identified by the study team on admission and screened against the inclusion/exclusion criteria.

Inclusion criteria

1. Gestational age ≤29w+6d (including infants born as one of a multiple birth)
2. Infants who receive respiratory support for at least 2 continuous hours' duration during the first 72 hours of life (intubated, or by non-invasive mechanical ventilation including continuous positive airway pressure and high flow nasal cannula or a combination thereof)
3. Presence of an indwelling intravenous line for drug administration
4. Written informed parental/guardian consent within 72 hours of birth

For peer review only - http://bmjopen.bmj.com/site/about/guidelines.xhtml

5. Anticipating administration of first dose within 72 hours of birth at the latest (optimally targeting within 24 hours after birth for inborn and 48 hours for outborn infants)
6. Reasonable expectancy to complete 10 days of trial treatment whilst resident at the recruiting site
7. Inborn, or born at site within the recruiting site's neonatal network where follow up will be possible
8. In the opinion of the local Principal Investigator, reasonable prospect of survival past the first 72 hours of life

Exclusion criteria

1. Exposure to another systemic macrolide antibiotic (not maternal)
2. Presence of major surgical or congenital abnormalities (not including patent ductus arteriosus or patent foramen ovale)
3. Contraindication of azithromycin as specified in the summary of characteristics of the product
4. Participation in other interventional trial that precludes participation in AZTEC

Trial intervention

The investigational medicinal product (IMP) is manufactured and QP released by Saint Mary's Pharmaceutical Unit (SMPU), Cardiff, UK (MIA[IMP]35929).

The dosing schedule is 20mg/kg (10mL/kg) azithromycin for 3 days, followed by 10mg/kg (5mL/kg) for 7 days, or placebo (10 days total). All doses are given via intravenous infusion (central or peripheral line) over a period of at least 1 hour. Azithromycin is most likely to have an effect when administered early to establish a sufficient concentration to eliminate *Ureaplasma*; a 20mg/kg dose for 3 days has recently been shown to be highly effective [22]. Treatment for a further 7 days is justified to treat the rise in pulmonary inflammation which peaks between 7-10 days after birth [9 25]. Sites have therefore been asked to target initiation of trial treatment at the earliest opportunity (and within 72 hours after birth at the latest).

Blinding

IMP is supplied as a patient pack of 12 blinded vials as illustrated in Figure 1 below. The vial blinding method utilises a custom cardboard carton sourced by SMPU, as used previously in a similar trial design [26]. Labelling was performed by SMPU as per a randomisation list provided by the Centre for Trials Research (CTR), Cardiff University.

For peer review only - http://bmjopen.bmj.com/site/about/guidelines.xhtml

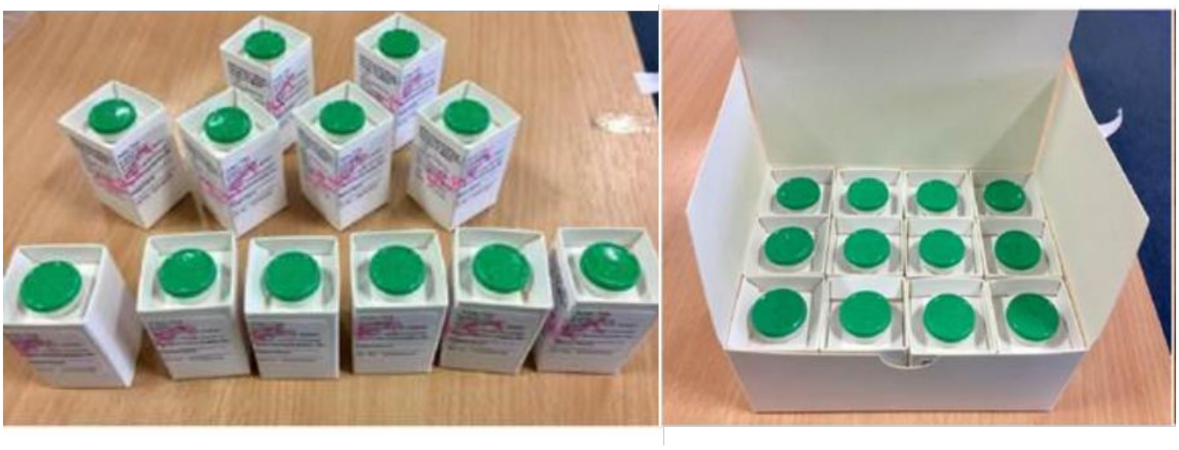

Figure 1: Example of AZTEC blinded IMP vials

For peer review only

Active arm

The active arm comprises of commercially-available 500mg vials of azithromycin powder for solution for infusion (Aspire Pharma Ltd). As per the summary of product characteristics (https://www.medicines.org.uk/emc/product/1276/smpc), 4.8mL sterile water is used to reconstitute the powder to obtain a clear, colourless solution of 100mg/ml. The administration concentration of 2mg/ml is obtained by withdrawing 1mL of solution from the vial and adding to 50mL of an acceptable diluent.

Control arm

The placebo arm is initially comprised of an empty, sterile vial manufactured to the same specification as the active product (released under GMP standards) and provided to SMPU by Aspire Pharma Ltd. for blinding and packaging. The same preparation steps are followed as per the active arm to produce a solution matched in appearance to the active arm.

Outcomes

Primary outcome

The primary outcome is a combined outcome of CLD (moderate-severe) and mortality at 36 weeks' PMA (or discharge, if sooner). The definition of CLD severity is based on consensus criteria [27], Table 1.

Table 1: Severity-based criteria for diagnosis of CLD at 36 weeks postmenstrual age

Received respiratory support and/or supplementary oxygen for **more than** 28 days, cumulatively, and the following:

For peer review only - http://bmjopen.bmj.com/site/about/guidelines.xhtml

Mild CLD	• Breathing room air
Moderate CLD	• Require <30% oxygen (or low flow 0.01-1.0 L/min), **not** receiving any respiratory support
Severe CLD	• Require ≥30% oxygen (or low flow ≥1.1 L/min), **still** receiving respiratory support (ventilation, CPAP, high flow oxygen)

Infants meeting the initial diagnosis of moderate CLD will undergo a physiological test to confirm their oxygen requirement (Figure 2). This physiological definition, initially developed by Quine and colleagues [28], has been widely used in clinical trials of neonatal lung disease [29].

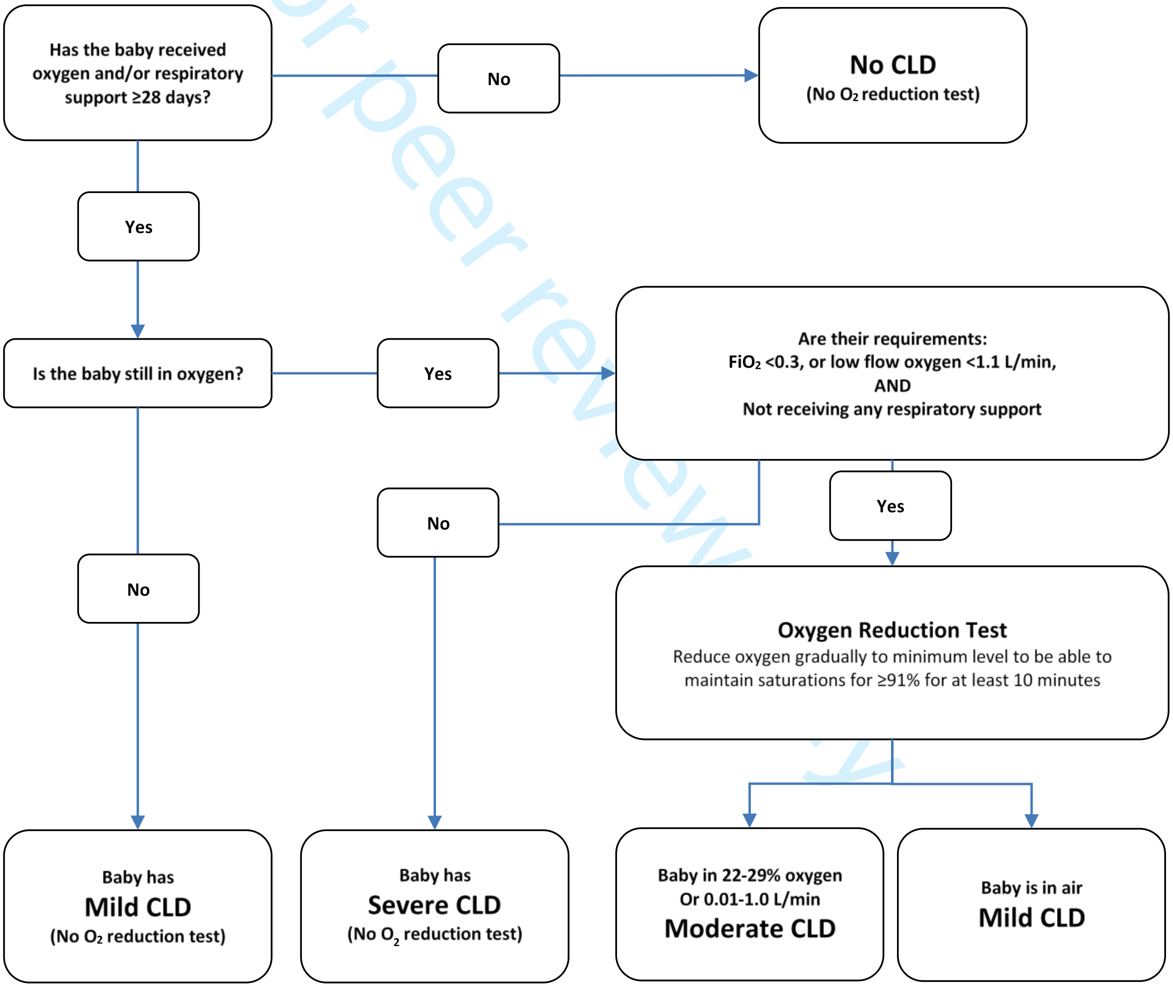

Figure 2: Flow diagram for assessment of CLD severity in the AZTEC trial. Modified from the original *https://www.npeu.ox.ac.uk/downloads/files/baby-oscar/protocol/Baby-OSCAR%20Protocol%20v6_171116.pdf*

Secondary outcomes will include:

1. Mortality rate by 36 weeks PMA
2. CLD severity by 36 weeks of age or discharge

For peer review only - http://bmjopen.bmj.com/site/about/guidelines.xhtml

3. Number of days of respiratory support/oxygen dependency
4. Development of complications of prematurity
    a. Nosocomial infection
    b. Severe intraventricular haemorrhage (grade III/IV)
    c. Necrotising enterocolitis (Bell stage II and above)
    d. Treatment for retinopathy of prematurity
    e. Treatment for patent ductus arteriosus
    f. Liver and renal function
5. Serious adverse events/reactions
6. Resistance to macrolides among microbes isolated from stool samples

Trial procedures

Site selection and training

Site selection is based on receipt of an expression of interest via the National Institute for Health Research networks and personal contact directly with UK Level 3 neonatal units. A registration questionnaire captures key information about the site, trial team, and any concerns around delivery of the protocol in the context of routine practice. Where feasible, a preliminary visit is made by the Trial Manager to present the study background and protocol. The Trial Management Group approved the centres selected to participate.

Formal initiation visits are held at each site to train the local trial team in trial-specific procedures. This is supplemented with comprehensive guidance documents for discrete elements of the study. Specific training in preparation and administration of the IMP is cascaded down by delegated members of the local study team to cot-side nurses; this includes a video produced by JL with support from neonatal nurses and the medical illustration department at Cardiff and Vale University Health Board (https://youtu.be/Xto6n5qFuYQ). The local clinical trials pharmacy team are also provided with trial-specific training in IMP storage, accountability and reconciliation procedures during the initiation process. All local study teams have undertaken Good Clinical Practice training commensurate with their roles and responsibilities.

Participant recruitment

The trial design is summarised in Figure 3 below. Recruitment is anticipated to take 30 months with 25 sites participating (an average of 1.5 infants per site, per month over the course of recruitment). Progress of the trial can be followed at www.aztec-trial.uk.

For peer review only - http://bmjopen.bmj.com/site/about/guidelines.xhtml

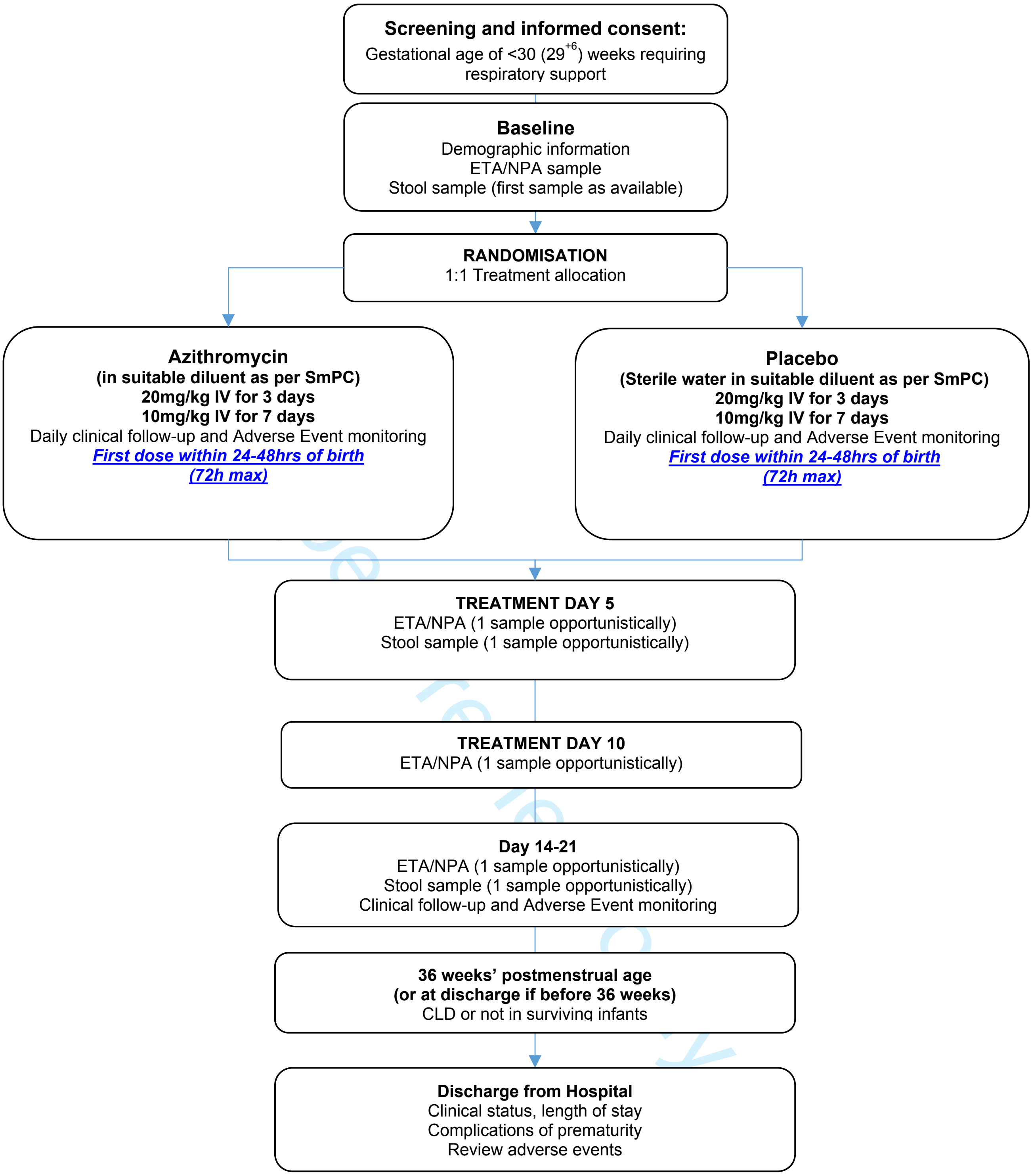

Figure 3: Flow diagram for AZTEC study procedures and follow-up. ETA= endotracheal aspirates, NPA= nasopharyngeal aspirates

For peer review only - http://bmjopen.bmj.com/site/about/guidelines.xhtml

Screening and consent

It is anticipated that all infants who meet the gestational age criteria for the trial (i.e. born <30 weeks' gestational age) are registered on the anonymised screening log. Reasons for exclusion, and reasons for not randomising otherwise eligible infants (e.g. declined consent) are being recorded.

A member of the local site team identifies potential eligible preterm babies antenatally with mothers with threatened preterm labour or babies admitted to the neonatal unit and the parent(s) receive a verbal description of the trial. Should the parent(s) express an interest, the ethically-approved information sheet and consent form are provided. Eligibility is confirmed by a medically qualified member of the trial team. Parents are given sufficient time to read the information and to ask questions during a further consultation with the study team. If the parents are willing to participate, they are asked to sign the consent form which is countersigned by the PI or a delegated member of the study team who engaged the parents in the informed consent discussion. Consent for use of samples in ancillary studies, and for future follow-up contact are optional. The right to withdraw from the study at any time without any affecting their baby's clinical care will be clearly communicated.

Randomisation

Randomisation to the intervention is performed following confirmation of eligibility and completion of the informed consent process. The randomisation list prepared by an independent CTR statistician uses a 1:1 allocation ratio with fixed block length. Allocation to azithromycin or placebo is blinded such that the allocation will not be known by clinicians, the baby's family or the trial outcome assessors. The list is uploaded to a validated, user tested, web-based system (Sortition™, Oxford University Innovation Ltd.). Randomisation is performed by a member of the local study team using a unique username and password. The system performs treatment allocation by issuing a random 4-digit IMP pack ID (matching the IMP supplied to each site), and also a participant ID in a standardised format. A member of the site study team retrieves the appropriate pack of IMP from the storage location and ensures this is appropriately prescribed on the infant's prescription chart. Unblinding, if required, may also be performed by the site PI (or designee) using the web-based system.

Data collection and sampling

Baseline/clinical assessments

Eligibility Case Report Forms (CRF) capture the required information to ensure infants meet the specified criteria. The focus of the baseline data collection is on maternal history and antenatal information, and details around the infant's birth.

For peer review only - http://bmjopen.bmj.com/site/about/guidelines.xhtml

Follow-up assessments

IMP administration, sample collection, use of intravenous antibiotics, and details of any positive blood/cerebrospinal fluid cultures are recorded on a daily basis until the infant is 21-days post randomisation. Should the infant be transferred from the AZTEC recruiting site to another hospital for continuation of their care, a transfer CRF will record summary data of their hospital stay. The receiving centre continues with follow-up assessments to support collection of primary and secondary outcomes.

Infants remain in follow up for safety and outcome purposes until 36 weeks' PMA, or discharge home if sooner (the last timepoint for recording new adverse reactions).

Sampling

Sites are collecting serial respiratory secretions and stool samples in a pragmatic, opportunistic manner around nominal timepoints of Baseline, day 5, day 10 and days 14-21 post-randomisation. These procedures are timed to mirror standard care to minimise disturbing trial infants, where possible. Endotracheal aspirates are obtained if the infant is intubated, and nasopharyngeal aspirates are collected if they are not. Stool samples are collected opportunistically whenever the infants open their bowls. All samples are refrigerated until shipment at the earliest opportunity overnight to the central laboratory where are be processed according to a standardised procedure and stored at -80°C pending analysis. A schematic of the sampling plan is show in Figure 4.

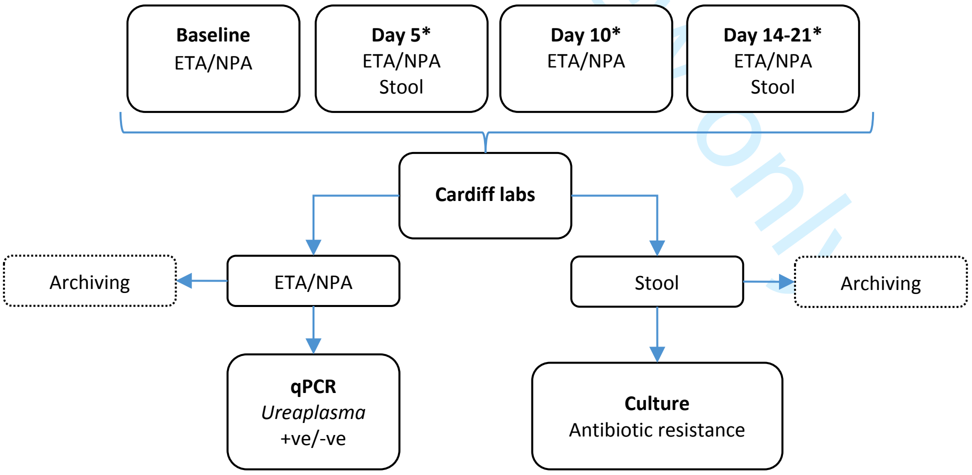

Figure 4: AZTEC biological sampling plan Day 4-6 is acceptable for the day 5 samples. Day 8-12 is acceptable for the day 10 sample. Day 14-21- one sample of each type during this period.*

For peer review only - http://bmjopen.bmj.com/site/about/guidelines.xhtml

Analysis

Sample size

Relevant interventional studies to prevent development of CLD as an outcome (including studies using macrolides) in preterm infants were reviewed. In general, national and international studies consistently show rates of survival without CLD of 50 – 60% (those with lower rates are due to highly selected groups of sicker participants) for studies with similar inclusion criteria to AZTEC. Thus, adopting a conservative approach using 50% survival without developing CLD is reasonable. Similarly, the absolute differences in effect size ranges from 10% to 20% in most studies. The study is powered to an improvement of 12% (50% to 62%) in survival without CLD with a power of 0.90 and significance level of 5% requiring recruitment of 796 subjects which is highly feasible as shown by several similar UK studies in this population. Since the primary outcome will involve formal assessment with an oxygen challenge test in both tertiary units and in step-down units, a dropout rate of 10% has been estimated.

Statistical analysis

We are conducting an internal pilot involving the first 5 sites to be activated, and for a period of 12 months (9 months of recruitment and 3 months of follow-up). A pilot report will be produced detailing information on recruitment rate, consent rates, treatment compliance and primary outcome completeness. The report will be presented to the funder and the AZTEC independent committees to help inform any adaptations necessary to facilitate moving forward with the main trial.

The trial will be analysed and reported using the 'Consolidated Standard of Reporting Trials' (CONSORT) [30] and the ICH E9 guidelines [31]. A separate and full statistical analysis plan has been developed prior to database lock. The analysis plan will be reviewed by the AZTEC independent committees.

The principle of intention-to-treat will be applied as far as practically possible including all participants in the primary analysis in the group to which they were randomly allocated. All analyses will use a 5% level of statistical significance and 95% confidence intervals will be presented throughout. The primary outcome will be analysed using two-level logistic regression (participants within centres) fitting treatment group and gestational age at birth fixed effects. The presence of multiple births within the same pregnancy will be explored by including this as an additional level in the model, and this will be used as the primary analytical approach should the model converge. Dichotomous secondary outcomes will be analysed using the same approach. Number of days of

For peer review only - http://bmjopen.bmj.com/site/about/guidelines.xhtml

respiratory support will be analysed as a time to event outcome allowing for competing risk of death. These models will also attempt to account for any clustering effects of multiple births within the same mother. Statistical tests will not be used on safety and tolerability outcomes. These outcomes will use descriptive statistics only.

To explore the extent to which there may be a differential treatment effect by presence of *Ureaplasma spp.*, the model fitted for the primary analysis will be extended by including a main and treatment group interaction term for *Ureaplasma* spp. colonisation at baseline. Since patterns of *Ureaplasma* colonisation may vary[32], the approach to this analysis may be modified in respect of emerging trends identified the placebo group samples. The final analytical approach will be agreed prior to database lock. Estimates from the statistical models (main effects and interaction terms) will be presented alongside 95% confidence intervals and p-values.

Microbiology

Respiratory samples are being processed via centrifugation to separate the supernatant and obtain a cell pellet from which DNA will be extracted. Identification *of Ureaplasma urealyticum*, *Ureaplasma parvum* and *Mycoplasma hominis* is being performed using multiplex quantitative polymerase chain reaction (qPCR) assay as previously described [33 34]. All DNA extractions and qPCR assays are being performed in collaboration with Central Biotechnology Services (Cardiff University, UK) according to standard operating procedures and GCP to minimise contamination.

Stool samples are being cultured aerobically and non-aerobically and in the presence and absence of azithromycin to assess baseline and new development of azithromycin-resistant organisms.

Discussion

As the survival of preterm infants increases, decreasing morbidity associated with prematurity including CLD is becoming increasingly important. AZTEC is an example of evaluating drug development [35] in infants as a large, pragmatic, adequately powered trial of a macrolide antibiotic to prevent development of CLD with widespread support by the neonatal community [36 37]. The importance of improving outcomes for preterm infants at risk of developing CLD is highlighted by the potential impacts on later pulmonary function, especially the prospect of early decline in lung function and early-onset COPD [38]. Moreover, the evidence base to treat graduates of the neonatal unit when they present with symptoms in childhood is poor [39], largely because the mechanisms of prematurity-associated respiratory morbidity remain poorly understood [40].

For peer review only - http://bmjopen.bmj.com/site/about/guidelines.xhtml

For peer review only

Whether modification of pulmonary inflammation and treatment of *Ureaplasma* infection would reduce rates of CLD has been debated for three decades. Use of postnatal corticosteroids is associated with long term adverse effects, however the safety profile of azithromycin is superior and is unlikely to affect neurological outcomes. Plans to follow up neurodevelopment and respiratory outcomes at one and two years of age are in place. This study includes ascertainment of whether each infant is colonised with *Ureaplasma* spp. and randomisation of treatment to azithromycin or placebo. Given the time lag between randomisation and the results of tests for *Ureaplasma* spp. the randomisation will not be balanced for *Ureaplasma* status. Nevertheless, this study design will allow an assessment of the causative role of *Ureaplasma* in the development of CLD.

If effectiveness of azithromycin therapy is demonstrated, integration into standard care would be relatively straightforward since the majority of candidates for treatment would have I.V. access for 7-10 days. Establishing whether azithromycin alters antibiotic resistance patterns will provide important information to the clinician who may have concerns around adding an adjunct treatment to a wide array of antimicrobials already given to this vulnerable group of patients. Although AZTEC will not specifically address mechanistic actions of azithromycin including its effect on the lung and gut microbiota, samples will be banked to permit such work in the near future using established [25], and novel methodologies such as metagenomics [41].

In summary, the AZTEC trial is addressing an important therapeutic need in an area where treatment based on high-quality evidence is severely lacking. Should antimicrobial resistance not be affected, azithromycin therapy should be appealing to clinicians due to the anti-inflammatory properties and proven ability to eradicate *Ureaplasma.* Treatment may yield fewer intensive care days and fewer discharges on home oxygen resulting in considerable reduction in costs to the NHS, as well as decreasing the significant burden on parents. On the other hand, should the treatment not be clinically effective the neonatal research community can turn their attention to alternative options for combatting CLD. Identification of an effective treatment to prevent CLD would be of exceptional value to the NHS, infants and their families

Ethics and dissemination

The current version of the AZTEC protocol is 3.0, dated 20/06/2020. Ethics permission has granted by the Wales Research Ethics Committee 2 (Ref 18/WA/0199), and regulatory permission by the Medicines and Healthcare Products Regulatory Agency (CTA reference 21323/0050/001-0001). NHS permission has been granted by the Health Research Authority and capacity and capability confirmed by each individual NHS organisation. The study is registered on EudraCT (2018-001109-99), ISRCTN (ISRCTN11650227) and on the National Institute for Health Research portfolio (CPMS

For peer review only - http://bmjopen.bmj.com/site/about/guidelines.xhtml

39385). Cardiff University is the Sponsor (resgov@cardiff.ac.uk), and were not involved in the preparation of this manuscript or the decision to submit. Personal data is held with the explicit consent of participants, independently of study data. The Centre for Trials Research has policies and procedures relating to data requests: https://www.cardiff.ac.uk/centre-for-trials-research/about-us/data-requests.

Oversight of the study is being performed by an independent Data Monitoring Committee and an independent Trial Steering Committee. Appointments to these committees were made with approval of the NIHR HRA.

Patient and public involvement

Parental input was obtained during the grant application on the design and conduct of the study. Parent representatives also reviewed public-facing information (e.g. information sheets and consent form) and are members of the Trial Management Group. An independent parent representative is a member of the Trial Steering Committee.

Authors contributions

SK is the Chief Investigator who developed the research question and secured funding together with JL, MT, and JB with subsequent input from JRM, NK, KH, DG, and ETJ. JL and SK wrote the protocol with input from all authors. JL is the Trial Manager, managed by ETJ Senior Trial Manager, and is responsible for coordinating the operational delivery of the protocol and recruitment. DG and TP are the trial statisticians and authored the statistical analysis plan. JL and NKi led the development of the case report forms; NKi supervises data management. LZ is the laboratory analyst; expert microbiology advice is provided by NK and JRM. SK, MT, MH and JB provide expert clinical input; MH provides nursing and clinical expertise. JL wrote the first draft of the manuscript supervised by SK. All authors provided critical review and final approval of the manuscript.

Funding statement

This work is supported by the National Institute of Health Research Health Technology Assessment programme (Ref 16/111/106). The study duration is 01/01/2019-30/09/2022.

For peer review only - http://bmjopen.bmj.com/site/about/guidelines.xhtml

Competing interests statement

SK declares research grant funding from the NIHR, MRC and JP Moulton Charitable Foundation for the submitted work; SK also declares personal fees from Aspire Pharma Ltd. JL, DG, LZ, ETJ, MT, NK, JRM and JB declare funding from the NIHR for the submitted work. The other authors have no competing interests to declare.

Acknowledgements

We are very grateful for the support provided from both the independent Data Monitoring Committee and Trial Steering Committee. The Centre for Trials Research receives core funding from the Welsh Government via Health & Care Research Wales and Cancer Research UK. We would like to acknowledge and thank the expert help we received from many parents and nursing staff to develop the initial application and protocol. In particular, we would like to thank the babies and their parents who have started to be enrolled to AZTEC.

For peer review only

For peer review only - http://bmjopen.bmj.com/site/about/guidelines.xhtml

References

1. Simpson SJ, Turkovic L, Wilson AC, et al. Lung function trajectories throughout childhood in survivors of very preterm birth: a longitudinal cohort study. *Lancet Child Adolesc Health* 2018;2(5):350-59. doi: 10.1016/s2352-4642(18)30064-6 [published Online First: 2018/09/01]
2. Edwards MO, Kotecha SJ, Lowe J, et al. Management of Prematurity-Associated Wheeze and Its Association with Atopy. *PLoS One* 2016;11(5):e0155695. doi: 10.1371/journal.pone.0155695 [published Online First: 2016/05/21]
3. Kotecha SJ, Edwards MO, Watkins WJ, et al. Effect of preterm birth on later FEV1: a systematic review and meta-analysis. *Thorax* 2013;68(8):760-6. doi: 10.1136/thoraxjnl-2012-203079 [published Online First: 2013/04/23]
4. Blencowe H, Cousens S, Oestergaard MZ, et al. National, regional, and worldwide estimates of preterm birth rates in the year 2010 with time trends since 1990 for selected countries: a systematic analysis and implications. *Lancet* 2012;379(9832):2162-72. doi: 10.1016/s0140-6736(12)60820-4 [published Online First: 2012/06/12]
5. Higgins RD, Jobe AH, Koso-Thomas M, et al. Bronchopulmonary Dysplasia: Executive Summary of a Workshop. *J Pediatr* 2018;197:300-08. doi: 10.1016/j.jpeds.2018.01.043 [published Online First: 2018/03/20]
6. Kotecha S. Lung growth for beginners. *Paediatr Respir Rev* 2000;1(4):308-13. doi: 10.1053/prrv.2000.0069 [published Online First: 2005/11/03]
7. Chakraborty M, McGreal EP, Kotecha S. Acute lung injury in preterm newborn infants: mechanisms and management. *Paediatr Respir Rev* 2010;11(3):162-70; quiz 70. doi: 10.1016/j.prrv.2010.03.002 [published Online First: 2010/08/10]
8. Abman SH, Bancalari E, Jobe A. The Evolution of Bronchopulmonary Dysplasia after 50 Years. *Am J Respir Crit Care Med* 2017;195(4):421-24. doi: 10.1164/rccm.201611-2386ED [published Online First: 2017/02/16]
9. Kotecha S, Chan B, Azam N, et al. Increase in interleukin-8 and soluble intercellular adhesion molecule-1 in bronchoalveolar lavage fluid from premature infants who develop chronic lung disease. *Arch Dis Child Fetal Neonatal Ed* 1995;72(2):F90-6. doi: 10.1136/fn.72.2.f90 [published Online First: 1995/03/01]
10. Maxwell NC, Davies PL, Kotecha S. Antenatal infection and inflammation: what's new? *Curr Opin Infect Dis* 2006;19(3):253-8. doi: 10.1097/01.qco.0000224819.42729.2e [published Online First: 2006/04/29]
11. Waites KB, Schelonka RL, Xiao L, et al. Congenital and opportunistic infections: Ureaplasma species and Mycoplasma hominis. *Semin Fetal Neonatal Med* 2009;14(4):190-9. doi: 10.1016/j.siny.2008.11.009 [published Online First: 2008/12/26]
12. Lowe J, Watkins WJ, Edwards MO, et al. Association between pulmonary ureaplasma colonization and bronchopulmonary dysplasia in preterm infants: updated systematic review and meta-analysis. *Pediatr Infect Dis J* 2014;33(7):697-702. doi: 10.1097/INF.0000000000000239 [published Online First: 2014/01/22]
13. Filippone M, Nardo D, Bonadies L, et al. Update on Postnatal Corticosteroids to Prevent or Treat Bronchopulmonary Dysplasia. *Am J Perinatol* 2019;36(S 02):S58-s62. doi: 10.1055/s-0039-1691802 [published Online First: 2019/06/27]
14. Mabanta CG, Pryhuber GS, Weinberg GA, et al. Erythromycin for the prevention of chronic lung disease in intubated preterm infants at risk for, or colonized or infected with Ureaplasma urealyticum. *Cochrane Database Syst Rev* 2003(4):CD003744. doi: 10.1002/14651858.CD003744 [published Online First: 2003/10/30]
15. Ozdemir R, Erdeve O, Dizdar EA, et al. Clarithromycin in preventing bronchopulmonary dysplasia in Ureaplasma urealyticum-positive preterm infants. *Pediatrics* 2011;128(6):e1496-501. doi: 10.1542/peds.2011-1350 [published Online First: 2011/11/30]

For peer review only - http://bmjopen.bmj.com/site/about/guidelines.xhtml

16. Alvarez-Elcoro S, Enzler MJ. The macrolides: erythromycin, clarithromycin, and azithromycin. *Mayo Clin Proc* 1999;74(6):613-34. doi: 10.4065/74.6.613 [published Online First: 1999/06/23]
17. Aghai ZH, Kode A, Saslow JG, et al. Azithromycin suppresses activation of nuclear factor-kappa B and synthesis of pro-inflammatory cytokines in tracheal aspirate cells from premature infants. *Pediatr Res* 2007;62(4):483-8. doi: 10.1203/PDR.0b013e318142582d [published Online First: 2007/08/02]
18. Legrand T, Elie V, Kotecha S, et al. An optimal LC-MS/MS method for determination of azithromycin in white blood cells: application to pediatric samples. *Bioanalysis* 2014;6(17):2317-28. doi: 10.4155/bio.14.81 [published Online First: 2014/11/11]
19. Nair V, Loganathan P, Soraisham AS. Azithromycin and other macrolides for prevention of bronchopulmonary dysplasia: a systematic review and meta-analysis. *Neonatology* 2014;106(4):337-47. doi: 10.1159/000363493 [published Online First: 2014/10/04]
20. Hassan HE, Othman AA, Eddington ND, et al. Pharmacokinetics, safety, and biologic effects of azithromycin in extremely preterm infants at risk for ureaplasma colonization and bronchopulmonary dysplasia. *J Clin Pharmacol* 2011;51(9):1264-75. doi: 10.1177/0091270010382021 [published Online First: 2010/11/26]
21. Merchan LM, Hassan HE, Terrin ML, et al. Pharmacokinetics, microbial response, and pulmonary outcomes of multidose intravenous azithromycin in preterm infants at risk for Ureaplasma respiratory colonization. *Antimicrob Agents Chemother* 2015;59(1):570-8. doi: 10.1128/AAC.03951-14 [published Online First: 2014/11/12]
22. Viscardi RM, Terrin ML, Magder LS, et al. Randomised trial of azithromycin to eradicate Ureaplasma in preterm infants. *Archives of Disease in Childhood - Fetal and Neonatal Edition* 2020:fetalneonatal-2019-318122. doi: 10.1136/archdischild-2019-318122
23. Smith C, Egunsola O, Choonara I, et al. Use and safety of azithromycin in neonates: a systematic review. *BMJ Open* 2015;5(12):e008194. doi: 10.1136/bmjopen-2015-008194 [published Online First: 2015/12/15]
24. Beeton ML, Chalker VJ, Jones LC, et al. Antibiotic Resistance among Clinical Ureaplasma Isolates Recovered from Neonates in England and Wales between 2007 and 2013. *Antimicrob Agents Chemother* 2016;60(1):52-6. doi: 10.1128/AAC.00889-15 [published Online First: 2015/10/16]
25. Gallacher D, Mitchell E, Alber D, et al. Dissimilarity of the gut-lung axis and dysbiosis of the lower airways in ventilated preterm infants. *Eur Respir J* 2020 doi: 10.1183/13993003.01909-2019 [published Online First: 2020/02/16]
26. Bruynseels D, Solomon C, Hallam A, et al. Commentary on Reconstituting Fibrinogen Concentrate to Maintain Blinding in a Double-blind, Randomized Trial in an Emergency Setting. *J Emerg Med* 2016;50(1):104-7 e1. doi: 10.1016/j.jemermed.2015.09.017 [published Online First: 2015/10/29]
27. Jobe AH, Bancalari E. Bronchopulmonary dysplasia. *Am J Respir Crit Care Med* 2001;163(7):1723-9. doi: 10.1164/ajrccm.163.7.2011060 [published Online First: 2001/06/13]
28. Quine D, Wong CM, Boyle EM, et al. Non-invasive measurement of reduced ventilation:perfusion ratio and shunt in infants with bronchopulmonary dysplasia: a physiological definition of the disease. *Arch Dis Child Fetal Neonatal Ed* 2006;91(6):F409-14. doi: 10.1136/adc.2006.095406 [published Online First: 2006/06/27]
29. The BOOST-II Australia and United Kingdom Collaborative Groups. Outcomes of Two Trials of Oxygen-Saturation Targets in Preterm Infants. *New England Journal of Medicine* 2016;374(8):749-60. doi: 10.1056/NEJMoa1514212
30. Moher D, Schulz KF, Altman D. The CONSORT statement: revised recommendations for improving the quality of reports of parallel-group randomized trials. *Jama* 2001;285(15):1987-91. doi: 10.1001/jama.285.15.1987 [published Online First: 2001/04/20]

For peer review only - http://bmjopen.bmj.com/site/about/guidelines.xhtml

31. International Council for Harmonisation. ICH E9: Statistical Principles for Clinical Trials, Step 5 1998 [Available from: https://www.ema.europa.eu/en/documents/scientific-guideline/ich-e-9-statistical-principles-clinical-trials-step-5_en.pdf accessed 17/04/2020 2020.
32. Castro-Alcaraz S, Greenberg EM, Bateman DA, et al. Patterns of colonization with Ureaplasma urealyticum during neonatal intensive care unit hospitalizations of very low birth weight infants and the development of chronic lung disease. *Pediatrics* 2002;110(4):e45. doi: 10.1542/peds.110.4.e45 [published Online First: 2002/10/03]
33. Payne MS, Goss KC, Connett GJ, et al. A quantitative analysis of Ureaplasma urealyticum and Ureaplasma parvum compared with host immune response in preterm neonates at risk of developing bronchopulmonary dysplasia. *J Clin Microbiol* 2012;50(3):909-14. doi: 10.1128/JCM.06625-11 [published Online First: 2011/12/23]
34. Férandon C, Peuchant O, Janis C, et al. Development of a real-time PCR targeting the yidC gene for the detection of Mycoplasma hominis and comparison with quantitative culture. *Clin Microbiol Infect* 2011;17(2):155-9. doi: 10.1111/j.1469-0691.2010.03217.x [published Online First: 2010/03/20]
35. Turner MA, Jacqz-Aigrain E, Kotecha S. Azithromycin, Ureaplasma and chronic lung disease of prematurity: a case study for neonatal drug development. *Arch Dis Child* 2012;97(6):573-7. doi: 10.1136/adc.2010.195180 [published Online First: 2011/06/24]
36. Maxwell NC, Nuttall D, Kotecha S. Does Ureaplasma spp. cause chronic lung disease of prematurity: ask the audience? *Early Hum Dev* 2009;85(5):291-6. doi: 10.1016/j.earlhumdev.2008.12.002 [published Online First: 2009/01/16]
37. Pansieri C, Pandolfini C, Elie V, et al. Ureaplasma, bronchopulmonary dysplasia, and azithromycin in European neonatal intensive care units: a survey. *Sci Rep* 2014;4:4076. doi: 10.1038/srep04076 [published Online First: 2014/02/13]
38. Stocks J, Hislop A, Sonnappa S. Early lung development: lifelong effect on respiratory health and disease. *Lancet Respir Med* 2013;1(9):728-42. doi: 10.1016/s2213-2600(13)70118-8 [published Online First: 2014/01/17]
39. Duijts L, van Meel ER, Moschino L, et al. European Respiratory Society guideline on long-term management of children with bronchopulmonary dysplasia. *Eur Respir J* 2020;55(1) doi: 10.1183/13993003.00788-2019 [published Online First: 2019/09/29]
40. Lowe J, Kotecha SJ, Kotecha S. Long Term Effects Following Extreme Prematurity: Respiratory Problems. In: Boyle EM, Cusack J, eds. Emerging Topics and Controversies in Neonatology. Switzerland: Springer 2020:351-66.
41. Mack I, Sharland M, Berkley JA, et al. Antimicrobial Resistance Following Azithromycin Mass Drug Administration: Potential Surveillance Strategies to Assess Public Health Impact. *Clin Infect Dis* 2020;70(7):1501-08. doi: 10.1093/cid/ciz893 [published Online First: 2019/10/22]

For peer review only

For peer review only - http://bmjopen.bmj.com/site/about/guidelines.xhtml

STANDARD PROTOCOL ITEMS: RECOMMENDATIONS FOR INTERVENTIONAL TRIALS

SPIRIT 2013 Checklist: Recommended items to address in a clinical trial protocol and related documents*

Section/item	Item No	Description	Addressed on page number
**Administrative information**			
Title	1	Descriptive title identifying the study design, population, interventions, and, if applicable, trial acronym	1
Trial registration	2a	Trial identifier and registry name. If not yet registered, name of intended registry	2
	2b	All items from the World Health Organization Trial Registration Data Set	Throughout
Protocol version	3	Date and version identifier	15
Funding	4	Sources and types of financial, material, and other support	15
Roles and responsibilities	5a	Names, affiliations, and roles of protocol contributors	1, 16
	5b	Name and contact information for the trial sponsor	15
	5c	Role of study sponsor and funders, if any, in study design; collection, management, analysis, and interpretation of data; writing of the report; and the decision to submit the report for publication, including whether they will have ultimate authority over any of these activities	15
	5d	Composition, roles, and responsibilities of the coordinating centre, steering committee, endpoint adjudication committee, data management team, and other individuals or groups overseeing the trial, if applicable (see Item 21a for data monitoring committee)	15, 16

For peer review only

**Introduction**			
Background and rationale	6a	Description of research question and justification for undertaking the trial, including summary of relevant studies (published and unpublished) examining benefits and harms for each intervention	3-4
	6b	Explanation for choice of comparators	3-4
Objectives	7	Specific objectives or hypotheses	5
Trial design	8	Description of trial design including type of trial (eg, parallel group, crossover, factorial, single group), allocation ratio, and framework (eg, superiority, equivalence, noninferiority, exploratory)	5
**Methods: Participants, interventions, and outcomes**			
Study setting	9	Description of study settings (eg, community clinic, academic hospital) and list of countries where data will be collected. Reference to where list of study sites can be obtained	5
Eligibility criteria	10	Inclusion and exclusion criteria for participants. If applicable, eligibility criteria for study centres and individuals who will perform the interventions (eg, surgeons, psychotherapists)	5-6
Interventions	11a	Interventions for each group with sufficient detail to allow replication, including how and when they will be administered	6-7
	11b	Criteria for discontinuing or modifying allocated interventions for a given trial participant (eg, drug dose change in response to harms, participant request, or improving/worsening disease)	N/A
	11c	Strategies to improve adherence to intervention protocols, and any procedures for monitoring adherence (eg, drug tablet return, laboratory tests)	9
	11d	Relevant concomitant care and interventions that are permitted or prohibited during the trial	N/A
Outcomes	12	Primary, secondary, and other outcomes, including the specific measurement variable (eg, systolic blood pressure), analysis metric (eg, change from baseline, final value, time to event), method of aggregation (eg, median, proportion), and time point for each outcome. Explanation of the clinical relevance of chosen efficacy and harm outcomes is strongly recommended	7-9
Participant timeline	13	Time schedule of enrolment, interventions (including any run-ins and washouts), assessments, and visits for participants. A schematic diagram is highly recommended (see Figure)	10

For peer review only - http://bmjopen.bmj.com/site/about/guidelines.xhtml

Sample size	14	Estimated number of participants needed to achieve study objectives and how it was determined, including clinical and statistical assumptions supporting any sample size calculations	12-13
Recruitment	15	Strategies for achieving adequate participant enrolment to reach target sample size	9

Methods: Assignment of interventions (for controlled trials)

Allocation:			
Sequence generation	16a	Method of generating the allocation sequence (eg, computer-generated random numbers), and list of any factors for stratification. To reduce predictability of a random sequence, details of any planned restriction (eg, blocking) should be provided in a separate document that is unavailable to those who enrol participants or assign interventions	11
Allocation concealment mechanism	16b	Mechanism of implementing the allocation sequence (eg, central telephone; sequentially numbered, opaque, sealed envelopes), describing any steps to conceal the sequence until interventions are assigned	11
Implementation	16c	Who will generate the allocation sequence, who will enrol participants, and who will assign participants to interventions	11
Blinding (masking)	17a	Who will be blinded after assignment to interventions (eg, trial participants, care providers, outcome assessors, data analysts), and how	11
	17b	If blinded, circumstances under which unblinding is permissible, and procedure for revealing a participant's allocated intervention during the trial	11

Methods: Data collection, management, and analysis

Data collection methods	18a	Plans for assessment and collection of outcome, baseline, and other trial data, including any related processes to promote data quality (eg, duplicate measurements, training of assessors) and a description of study instruments (eg, questionnaires, laboratory tests) along with their reliability and validity, if known. Reference to where data collection forms can be found, if not in the protocol	11-12
	18b	Plans to promote participant retention and complete follow-up, including list of any outcome data to be collected for participants who discontinue or deviate from intervention protocols	11-12

Data management	19	Plans for data entry, coding, security, and storage, including any related processes to promote data quality (eg, double data entry; range checks for data values). Reference to where details of data management procedures can be found, if not in the protocol	11-12
Statistical methods	20a	Statistical methods for analysing primary and secondary outcomes. Reference to where other details of the statistical analysis plan can be found, if not in the protocol	13
	20b	Methods for any additional analyses (eg, subgroup and adjusted analyses)	13
	20c	Definition of analysis population relating to protocol non-adherence (eg, as randomised analysis), and any statistical methods to handle missing data (eg, multiple imputation)	13
**Methods: Monitoring**			
Data monitoring	21a	Composition of data monitoring committee (DMC); summary of its role and reporting structure; statement of whether it is independent from the sponsor and competing interests; and reference to where further details about its charter can be found, if not in the protocol. Alternatively, an explanation of why a DMC is not needed	15
	21b	Description of any interim analyses and stopping guidelines, including who will have access to these interim results and make the final decision to terminate the trial	13
Harms	22	Plans for collecting, assessing, reporting, and managing solicited and spontaneously reported adverse events and other unintended effects of trial interventions or trial conduct	11
Auditing	23	Frequency and procedures for auditing trial conduct, if any, and whether the process will be independent from investigators and the sponsor	15
**Ethics and dissemination**			
Research ethics approval	24	Plans for seeking research ethics committee/institutional review board (REC/IRB) approval	15
Protocol amendments	25	Plans for communicating important protocol modifications (eg, changes to eligibility criteria, outcomes, analyses) to relevant parties (eg, investigators, REC/IRBs, trial participants, trial registries, journals, regulators)	15

For peer review only - http://bmjopen.bmj.com/site/about/guidelines.xhtml

Consent or assent	26a	Who will obtain informed consent or assent from potential trial participants or authorised surrogates, and how (see Item 32)	11
	26b	Additional consent provisions for collection and use of participant data and biological specimens in ancillary studies, if applicable	11
Confidentiality	27	How personal information about potential and enrolled participants will be collected, shared, and maintained in order to protect confidentiality before, during, and after the trial	15
Declaration of interests	28	Financial and other competing interests for principal investigators for the overall trial and each study site	16
Access to data	29	Statement of who will have access to the final trial dataset, and disclosure of contractual agreements that limit such access for investigators	15
Ancillary and post-trial care	30	Provisions, if any, for ancillary and post-trial care, and for compensation to those who suffer harm from trial participation	NA
Dissemination policy	31a	Plans for investigators and sponsor to communicate trial results to participants, healthcare professionals, the public, and other relevant groups (eg, via publication, reporting in results databases, or other data sharing arrangements), including any publication restrictions	15
	31b	Authorship eligibility guidelines and any intended use of professional writers	NA
	31c	Plans, if any, for granting public access to the full protocol, participant-level dataset, and statistical code	15
**Appendices**			
Informed consent materials	32	Model consent form and other related documentation given to participants and authorised surrogates	NA
Biological specimens	33	Plans for collection, laboratory evaluation, and storage of biological specimens for genetic or molecular analysis in the current trial and for future use in ancillary studies, if applicable	12

*It is strongly recommended that this checklist be read in conjunction with the SPIRIT 2013 Explanation & Elaboration for important clarification on the items. Amendments to the protocol should be tracked and dated. The SPIRIT checklist is copyrighted by the SPIRIT Group under the Creative Commons "Attribution-NonCommercial-NoDerivs 3.0 Unported" license.

BMJ Open

Study Protocol: Azithromycin Therapy for Chronic Lung Disease of Prematurity (AZTEC): A randomised, placebo-controlled trial of azithromycin for the prevention of chronic lung disease of prematurity in preterm infants

Journal:	*BMJ Open*
Manuscript ID	bmjopen-2020-041528.R1
Article Type:	Protocol
Date Submitted by the Author:	31-Jul-2020
Complete List of Authors:	Lowe, John; Centre for Trials Research, University of Cardiff<br>Gillespie, David; Centre for Trials Research, University of Cardiff ,<br>Hubbard, Marie; University Hospitals of Leicester NHS Trust<br>Zhang, Lei; Cardiff University School of Medicine,<br>Kirby, Nigel; Cardiff University, Centre for Trials Research<br>Pickles, Timothy; Cardiff University, South East Wales Trials Unit (SEWTU)<br>Thomas-Jones, Emma; Cardiff University, Centre for Trials Research<br>Turner, Mark; Liverpool Women's Hospital NHS Foundation Trust,<br>Klein, Nigel; University College London<br>Marchesi, Julian; Cardiff University, School of Biosciences; Imperial College London, Department of Metabolism, Digestion and Reproduction, Faculty of Medicine<br>Hood, Kerenza; Cardiff University,<br>Berrington, Janet; Newcastle Upon Tyne Hospitals NHS Foundation Trust<br>Kotecha, Sailesh; Cardiff University School of Medicine, Department of Child Health, Institute of Molecular & Experimental Medicine
**Primary Subject Heading**:	Respiratory medicine
Secondary Subject Heading:	Paediatrics
Keywords:	NEONATOLOGY, Paediatric thoracic medicine < PAEDIATRICS, Chronic airways disease < THORACIC MEDICINE

SCHOLARONE™
Manuscripts

I, the Submitting Author has the right to grant and does grant on behalf of all authors of the Work (as defined in the below author licence), an exclusive licence and/or a non-exclusive licence for contributions from authors who are: i) UK Crown employees; ii) where BMJ has agreed a CC-BY licence shall apply, and/or iii) in accordance with the terms applicable for US Federal Government officers or employees acting as part of their official duties; on a worldwide, perpetual, irrevocable, royalty-free basis to BMJ Publishing Group Ltd ("BMJ") its licensees and where the relevant Journal is co-owned by BMJ to the co-owners of the Journal, to publish the Work in this journal and any other BMJ products and to exploit all rights, as set out in our licence.

The Submitting Author accepts and understands that any supply made under these terms is made by BMJ to the Submitting Author unless you are acting as an employee on behalf of your employer or a postgraduate student of an affiliated institution which is paying any applicable article publishing charge ("APC") for Open Access articles. Where the Submitting Author wishes to make the Work available on an Open Access basis (and intends to pay the relevant APC), the terms of reuse of such Open Access shall be governed by a Creative Commons licence – details of these licences and which Creative Commons licence will apply to this Work are set out in our licence referred to above.

Other than as permitted in any relevant BMJ Author's Self Archiving Policies, I confirm this Work has not been accepted for publication elsewhere, is not being considered for publication elsewhere and does not duplicate material already published. I confirm all authors consent to publication of this Work and authorise the granting of this licence.

For peer review only

Study Protocol: Azithromycin Therapy for Chronic Lung Disease of Prematurity (AZTEC): A randomised, placebo-controlled trial of azithromycin for the prevention of chronic lung disease of prematurity in preterm infants

John Lowe[1], David Gillespie[1], Marie Hubbard[2], Lei Zhang[3], Nigel Kirby[1], Tim Pickles[1], Emma Thomas-Jones[1], Mark Turner[4], Nigel Klein[5], Julian Marchesi[6], Kerenza Hood[1], Janet Berrington[7], Sailesh Kotecha[3]

1 Centre for Trials Research, College of Biomedical and Life Sciences, Cardiff University, Cardiff, UK

2 University Hospitals of Leicester NHS Foundation Trust, Leicester, UK

3 Department of Child Health, School of Medicine, Cardiff University, Cardiff, UK

4 Women and Children's Health, University of Liverpool, UK

5 GOS Institute of Child Health, University College London, London, UK

6 School of Biosciences, Cardiff University, Cardiff, UK

7 The Newcastle upon Tyne Hospitals NHS Foundation Trust, Newcastle upon Tyne, UK

Corresponding Author: Professor Sailesh Kotecha

Department of Child Health

School of Medicine

Cardiff University

Heath Park

Cardiff

CF14 4XN

United Kingdom

Email: KotechaS@cardiff.ac.uk

Telephone: 029 20 74 4187

Fax: 029 20 74 4283

For peer review only - http://bmjopen.bmj.com/site/about/guidelines.xhtml

Abstract

Introduction

Chronic Lung Disease of Prematurity (CLD), also known as Bronchopulmonary dysplasia (BPD), is a cause of significant respiratory morbidity in childhood and beyond. Coupled with lung immaturity, infections (especially by *Ureaplasma* spp.) are implicated in the pathogenesis of CLD through promotion of pulmonary inflammation. Azithromycin, which is a highly effective against *Ureaplasma* spp. also has potent anti-inflammatory properties. Thus, azithromycin therapy may improve respiratory outcomes by targeting infective and inflammatory pathways. Previous trials using macrolides have not been sufficiently powered to definitively assess CLD rates. To address this, the Azithromycin Therapy for Chronic Lung Disease (AZTEC) trial aims to determine if a 10-day early course of intravenous azithromycin improves rates of survival without CLD when compared to placebo with an appropriately powered study.

Methods and Analysis

796 infants born at less than 30 weeks' gestational age who require at least 2 hours of continuous respiratory support within the first 72 hours following birth are being enrolled by neonatal units in the UK. They are being randomised to receive a double-blind, once daily dose of intravenous azithromycin (20mg/kg for 3 days, followed by 10mg/kg for a further 7 days), or placebo. CLD is being assessed at 36 weeks' postmenstrual age. Whether colonisation with *Ureaplasma* spp. prior to randomisation modifies the treatment effect of azithromycin compared to placebo will also be investigated. Secondary outcomes include necrotising enterocolitis, intraventricular/cerebral haemorrhage, retinopathy of prematurity and nosocomial infections), development of antibiotic resistance; adverse reactions will be monitored.

Ethics and dissemination:

Ethics permission has been granted by Wales Research Ethics Committee 2 (Ref 18/WA/0199), and regulatory permission by the Medicines and Healthcare Products Regulatory Agency (CTA reference 21323/0050/001-0001). The study is registered on ISRCTN (ISRCTN11650227). The study is overseen by an independent Data Monitoring Committee and an independent Trial Steering Committee. We shall disseminate our findings via national and international peer-reviewed journals, and conferences. A summary of the findings will also be posted on the trial website.

For peer review only - http://bmjopen.bmj.com/site/about/guidelines.xhtml

Strengths and limitations of this study

- This is a randomised, double-blind placebo-controlled trial assessing an important outcome, and using an intervention which could be readily implemented in to clinical practice
- If the assumptions underlying the sample size calculation are valid, this study will be appropriately powered for the primary outcome
- The study will also help address the role of pulmonary *Ureaplasma* spp. colonisation in the development of CLD
- Universal administration of azithromycin may alter antibiotic resistance patterns, which are also being investigated in this study
- Further work will be required to investigate the mechanistic action of azithromycin including on the lung and gut microbiota

Introduction

Chronic Lung Disease of Prematurity (CLD), also known as Bronchopulmonary dysplasia (BPD), is a major cause of neonatal death in infants born prematurely. It is clear that survivors of CLD have adverse respiratory outcomes in childhood and beyond [1-3].

Despite the advances in neonatal care, rates of CLD have not markedly changed. This is largely because the survival of the most immature infants born at the limits viability (22 – 23 weeks' gestation) means that the proportion of preterm infants born at 28 weeks of less of gestation has increased over the last thirty years [4]. Consequently, the etiological definition of CLD was modified to represent the structural immaturity of the lungs which are in the canalicular/saccular stages of development [5 6]. The pathophysiology is complex and results from continual lung injury caused by inflammatory processes coupled with repair/remodelling and resultant fibrosis [7]. In contrast, the historical picture of CLD was largely characterised by parenchymal tissue damage caused by volutrauma, barotrauma and by high levels of oxygen required to sustain life [8].

Infections, both antenatal (e.g. chorioamnionitis) and nosocomial contribute to a cytokine-mediated inflammatory cascade which peaks between 7-10 days of life [9 10]. *Ureaplasma* spp. (class Mollicutes) are the smallest free living organisms and have long been implicated in the pathogenesis of CLD [11]. These commensal bacteria form part of the normal vaginal bacteria microbiota thus are readily transmitted to the uterus via the cervix or acquired during vaginal delivery. Lacking a cell wall, they are readily transferred to the lung where they colonise the respiratory mucosa and directly, or, in

For peer review only - http://bmjopen.bmj.com/site/about/guidelines.xhtml

conjunction with aforementioned iatrogenic factors, indirectly promote inflammation largely through recruitment of neutrophils to the lung. Our recent systematic review and meta-analysis reported that *Ureaplasma* spp. colonisation was strongly associated with diagnosis of CLD at 36 weeks' postmenstrual age (odds ratio 2.22; 95% CI 1.42, 3.47) which was largely independent of gestational age when investigated with meta-regression [12].

Current treatments for the prevention of CLD are largely supportive and include optimising use of early non-invasive respiratory support and preventing infections. Use of systemic corticosteroids is considered in cases where the infant is unable to be weaned from invasive ventilation. However, although early low-dose regimens may be beneficial in a high-risk subpopulation, it is well-established that higher doses of corticosteroids is associated with adverse neurodevelopmental outcomes including cerebral palsy [13].

Due to the potential risks associated with use of corticosteroids, other treatments, or ideally preventive strategies, with improved safety profiles are urgently required for CLD. An interest in macrolide antibiotics, and the potential for improving lung outcomes via eradication of *Ureaplasma* was established in the 1990s. However, early trials of erythromycin were underpowered and did not reduce the incidence or severity of CLD [14]. A study trialling clarithromycin treatment for 10 days reported a reduction in CLD (2.9% Vs. 36%) when compared to placebo but importantly only initiated delayed randomised therapy if the infant was culture-positive for *Ureaplasma* [15]. More contemporary data exists with azithromycin which is an important adjunct therapy for numerous respiratory conditions, including cystic fibrosis and chronic obstructive pulmonary disease (COPD). Consistent with other macrolides, azithromycin inhibits bacterial protein synthesis through blinding to the 50s bacterial ribosomal subunit [16]. Moreover, azithromycin is attractive as it also uniquely exhibits well-characterised immunomodulatory effects through suppression of Nuclear factor-kappaB, decreasing neutrophilic pulmonary inflammation by limiting the production of pro-inflammatory cytokines such as IL-6 and IL-8 [17]. Furthermore, since azithromycin is concentrated in leukocytes, it may be actively transported and released at the site of infection [18].

Several proof of concept and dose-finding studies have already been completed. The meta-analysis by Nair and colleagues showed that azithromycin treatment is associated with a 14% reduction in the combined outcome of CLD/death when compared to placebo [19]. Dose-finding work in respect of *Ureaplasma* spp. eradication has been undertaken in a series of studies by Viscardi and colleagues [20-22]. Importantly, all previous studies did not note any serious adverse reactions to azithromycin and add to the existing safety information [23]. Antimicrobial resistance amongst *Ureaplasma* remains low [24].

For peer review only - http://bmjopen.bmj.com/site/about/guidelines.xhtml

Given the Nair meta-analyses and lack of adequately powered studies, and given the reported association of presence of pulmonary inflammation and *Ureaplasma* with development of CLD, there is a compelling case for a definitive, adequately powered study to investigate the effectiveness of azithromycin on reducing rates of CLD in preterm infants. The Azithromycin Therapy for Chronic Lung Disease trial (AZTEC) is determining if a 10-day course of intravenous azithromycin improves rates of survival without CLD at 36 weeks' postmenstrual age when compared to placebo.

Methods and analysis

Primary Objective

The primary objective of the AZTEC trial is to assess the effectiveness of a 10-day course of azithromycin on improving survival without physiologically-defined CLD in infants born at <30 weeks' gestational age.

Design

AZTEC is a double-blind, randomised, placebo-controlled trial. The total samples size is 796 preterm-born infants; each are randomised individually (including infants from a multiple birth). Follow-up is undertaken at 36 weeks' post-menstrual age (PMA) to assess CLD status at 36 weeks' postmenstrual age, with final follow up a discharge from hospital.

Setting

Infants are being enrolled from UK tertiary neonatal units which are designated Level 3 (regional neonatal intensive care units), and followed up at their local hospital if transferred. Infants are identified by the study team on admission and screened against the inclusion/exclusion criteria.

Inclusion criteria

1. Gestational age ≤29w+6d (including infants born as one of a multiple birth)
2. Infants who receive respiratory support for at least 2 continuous hours' duration during the first 72 hours of life (intubated, or by non-invasive mechanical ventilation including continuous positive airway pressure and high flow nasal cannula or a combination thereof)
3. Presence of an indwelling intravenous line for drug administration
4. Written informed parental/guardian consent within 72 hours of birth

For peer review only - http://bmjopen.bmj.com/site/about/guidelines.xhtml

5. Anticipating administration of first dose within 72 hours of birth at the latest (optimally targeting within 24 hours after birth for inborn and 48 hours for outborn infants)
6. Reasonable expectancy to complete 10 days of trial treatment whilst resident at the recruiting site
7. Inborn, or born at site within the recruiting site's neonatal network where follow up will be possible
8. In the opinion of the local Principal Investigator, reasonable prospect of survival past the first 72 hours of life

Exclusion criteria

1. Exposure to another systemic macrolide antibiotic (not maternal)
2. Presence of major surgical or congenital abnormalities (not including patent ductus arteriosus or patent foramen ovale)
3. Contraindication of azithromycin as specified in the summary of characteristics of the product
4. Participation in other interventional trial that precludes participation in AZTEC

Trial intervention

The investigational medicinal product (IMP) is manufactured and QP released by Saint Mary's Pharmaceutical Unit (SMPU), Cardiff, UK (MIA[IMP]35929).

The dosing schedule is 20mg/kg (10mL/kg) azithromycin for 3 days, followed by 10mg/kg (5mL/kg) for 7 days, or placebo (10 days total). All doses are given via intravenous infusion (central or peripheral line) over a period of at least 1 hour. Azithromycin is most likely to have an effect when administered early to establish a sufficient concentration to eliminate *Ureaplasma*; a 20mg/kg dose for 3 days has recently been shown to be highly effective [22]. Treatment for a further 7 days is justified to treat the rise in pulmonary inflammation which peaks between 7-10 days after birth [9 25]. Sites have therefore been asked to target initiation of trial treatment at the earliest opportunity (and within 72 hours after birth at the latest).

Blinding

IMP is supplied as a patient pack of 12 blinded vials. The vial blinding method utilises a custom cardboard carton sourced by SMPU, as used previously in a similar trial design [26]. Labelling was performed by SMPU as per a randomisation list provided by the Centre for Trials Research (CTR), Cardiff University.

For peer review only - http://bmjopen.bmj.com/site/about/guidelines.xhtml

Active arm

The active arm comprises of commercially-available 500mg vials of azithromycin powder for solution for infusion (Aspire Pharma Ltd). As per the summary of product characteristics (https://www.medicines.org.uk/emc/product/1276/smpc), 4.8mL sterile water is used to reconstitute the powder to obtain a clear, colourless solution of 100mg/ml. The administration concentration of 2mg/ml is obtained by withdrawing 1mL of solution from the vial and adding to 50mL of an acceptable diluent.

Control arm

The placebo arm is initially comprised of an empty, sterile vial manufactured to the same specification as the active product (released under GMP standards) and provided to SMPU by Aspire Pharma Ltd. for blinding and packaging. The same preparation steps are followed as per the active arm to produce a solution matched in appearance to the active arm.

Outcomes

Primary outcome

The primary outcome is a combined outcome of CLD (moderate-severe) and mortality at 36 weeks' PMA (or discharge, if sooner). The definition of CLD severity is based on consensus criteria [27], Table 1.

Table 1: Severity-based criteria for diagnosis of CLD at 36 weeks postmenstrual age

Received respiratory support and/or supplementary oxygen for **more than** 28 days, cumulatively, and the following:	
Mild CLD	• Breathing room air
Moderate CLD	• Require <30% oxygen (or low flow 0.01-1.0 L/min), **not** receiving any respiratory support
Severe CLD	• Require ≥30% oxygen (or low flow ≥1.1 L/min), **still** receiving respiratory support (ventilation, CPAP, high flow oxygen)

Infants meeting the initial diagnosis of moderate CLD will undergo a physiological test to confirm their oxygen requirement (Figure 1). This physiological definition, initially developed by Quine and colleagues [28], has been widely used in clinical trials of neonatal lung disease [29].

Secondary outcomes will include:

1. Mortality rate by 36 weeks PMA

For peer review only - http://bmjopen.bmj.com/site/about/guidelines.xhtml

2. CLD severity by 36 weeks of age or discharge
3. Number of days of respiratory support/oxygen dependency
4. Development of complications of prematurity
    a. Nosocomial infection
    b. Severe intraventricular haemorrhage (grade III/IV)
    c. Necrotising enterocolitis (Bell stage II and above)
    d. Treatment for retinopathy of prematurity
    e. Treatment for patent ductus arteriosus
    f. Liver and renal function
5. Serious adverse events/reactions
6. Resistance to macrolides among microbes isolated from stool samples

Trial procedures

Site selection and training

Site selection is based on receipt of an expression of interest via the National Institute for Health Research networks and personal contact directly with UK Level 3 neonatal units. A registration questionnaire captures key information about the site, trial team, and any concerns around delivery of the protocol in the context of routine practice. Where feasible, a preliminary visit is made by the Trial Manager to present the study background and protocol. The Trial Management Group approved the centres selected to participate.

Formal initiation visits are held at each site to train the local trial team in trial-specific procedures. This is supplemented with comprehensive guidance documents for discrete elements of the study. Specific training in preparation and administration of the IMP is cascaded down by delegated members of the local study team to cot-side nurses; this includes a video produced by JL with support from neonatal nurses and the medical illustration department at Cardiff and Vale University Health Board (https://youtu.be/Xto6n5qFuYQ). The local clinical trials pharmacy team are also provided with trial-specific training in IMP storage, accountability and reconciliation procedures during the initiation process. All local study teams have undertaken Good Clinical Practice training commensurate with their roles and responsibilities.

Participant recruitment

The trial design is summarised in Figure 2. Recruitment is anticipated to take 30 months with 25 sites participating (an average of 1.5 infants per site, per month over the course of recruitment). Progress of the trial can be followed at www.aztec-trial.uk.

For peer review only - http://bmjopen.bmj.com/site/about/guidelines.xhtml

Screening and consent

It is anticipated that all infants who meet the gestational age criteria for the trial (i.e. born <30 weeks' gestational age) are registered on the anonymised screening log. Reasons for exclusion, and reasons for not randomising otherwise eligible infants (e.g. declined consent) are being recorded.

A member of the local site team identifies potential eligible preterm babies antenatally with mothers with threatened preterm labour or babies admitted to the neonatal unit and the parent(s) receive a verbal description of the trial. Should the parent(s) express an interest, the ethically-approved information sheet and consent form are provided (see supplementary file). Eligibility is confirmed by a medically qualified member of the trial team. Parents are given sufficient time to read the information and to ask questions during a further consultation with the study team. If the parents are willing to participate, they are asked to sign the consent form which is countersigned by the PI or a delegated member of the study team who engaged the parents in the informed consent discussion. Consent for use of samples in ancillary studies, and for future follow-up contact are optional. The right to withdraw from the study at any time without any affecting their baby's clinical care will be clearly communicated.

Randomisation

Randomisation to the intervention is performed following confirmation of eligibility and completion of the informed consent process. The randomisation list prepared by an independent CTR statistician uses a 1:1 allocation ratio with fixed block length. Allocation to azithromycin or placebo is blinded such that the allocation will not be known by clinicians, the baby's family or the trial outcome assessors. The list is uploaded to a validated, user tested, web-based system (Sortition™, Oxford University Innovation Ltd.). Randomisation is performed by a member of the local study team using a unique username and password. The system performs treatment allocation by issuing a random 4-digit IMP pack ID (matching the IMP supplied to each site), and also a participant ID in a standardised format. A member of the site study team retrieves the appropriate pack of IMP from the storage location and ensures this is appropriately prescribed on the infant's prescription chart. Unblinding, if required, may also be performed by the site PI (or designee) using the web-based system.

Data collection and sampling

Clinical assessments

Following documentation of eligibility, the focus of the baseline data collection is on maternal history and antenatal information, and details around the infant's birth.

For peer review only - http://bmjopen.bmj.com/site/about/guidelines.xhtml

IMP administration, sample collection, use of intravenous antibiotics, and details of any positive blood/cerebrospinal fluid cultures are recorded on a daily basis until the infant is 21-days post randomisation. Liver and kidney function will be monitored through collection of standard laboratory values. Should the infant be transferred from the AZTEC recruiting site to another hospital for continuation of their care, he receiving centre continues with follow-up assessments to support collection of primary and secondary outcomes.

Infants remain in follow up for safety and outcome purposes until 36 weeks' PMA, or discharge home if sooner (the last timepoint for recording new adverse reactions).

Data collection

All data are recorded on the trial electronic Case Report Form. Accumulating data are being regularly monitored and queries raised with sites should values be missing, or otherwise erroneous (e.g. validation against pre-specified ranges for laboratory values).

Sampling

Sites are collecting serial respiratory secretions and stool samples in a pragmatic, opportunistic manner around nominal timepoints of Baseline, day 5, day 10 and days 14-21 post-randomisation. These procedures are timed to mirror standard care to minimise disturbing trial infants, where possible. Endotracheal aspirates are obtained if the infant is intubated, and nasopharyngeal aspirates are collected if they are not. Stool samples are collected opportunistically whenever the infants open their bowls. All samples are refrigerated until shipment at the earliest opportunity overnight to the central laboratory where are be processed according to a standardised procedure and stored at -80°C pending analysis. A schematic of the sampling plan is show in Figure 3Figure .

Analysis

Sample size

Relevant interventional studies to prevent development of CLD as an outcome (including studies using macrolides) in preterm infants were reviewed. In general, national and international studies consistently show rates of survival without CLD of 50 – 60% (those with lower rates are due to highly selected groups of sicker participants) for studies with similar inclusion criteria to AZTEC. Thus, adopting a conservative approach using 50% survival without developing CLD is reasonable. Similarly, the absolute differences in effect size ranges from 10% to 20% in most studies. The study is powered to an improvement of 12% (50% to 62%) in survival without CLD with a power of 0.90 and significance level of 5% requiring recruitment of 796 subjects which is highly feasible as shown by

For peer review only - http://bmjopen.bmj.com/site/about/guidelines.xhtml

For peer review only

several similar UK studies in this population. Since the primary outcome will involve formal assessment with an oxygen challenge test in both tertiary units and in step-down units, a dropout rate of 10% has been estimated.

Statistical analysis

We are conducting an internal pilot involving the first 5 sites to be activated, and for a period of 12 months (9 months of recruitment and 3 months of follow-up). A pilot report will be produced detailing information on recruitment rate, consent rates, treatment compliance and primary outcome completeness. The report will be presented to the funder and the AZTEC independent committees to help inform any adaptations necessary to facilitate moving forward with the main trial. No formal interim analysis is planned.

The trial will be analysed and reported using the 'Consolidated Standard of Reporting Trials' (CONSORT) [30] and the ICH E9 guidelines [31]. A separate and full statistical analysis plan has been developed prior to database lock. The analysis plan will be reviewed by the AZTEC independent committees.

The principle of intention-to-treat will be applied as far as practically possible including all participants in the primary analysis in the group to which they were randomly allocated. All analyses will use a 5% level of statistical significance and 95% confidence intervals will be presented throughout. The primary outcome will be analysed using multilevel logistic regression, within a multiple imputation framework. The analysis will adjust for covariates balanced on at randomisation and account for both clustering of both multiple births and participants within centres. The imputation model will use the treatment arm and gestational age variables as well as whether or not the participant was transferred from their recruiting site prior to the primary outcome assessment. Furthermore, a series of sensitivity analyses, within the multiple imputation framework, will be used to assess the robustness of conclusions.

The presence of multiple births within the same pregnancy will be explored by including this as an additional level in the model, and this will be used as the primary analytical approach should the model converge. Dichotomous secondary outcomes will be analysed using the same approach. Number of days of respiratory support will be analysed as a time to event outcome allowing for competing risk of death. These models will also attempt to account for any clustering effects of multiple births within the same mother. Statistical tests will not be used on safety and tolerability outcomes. These outcomes will use descriptive statistics only.

For peer review only - http://bmjopen.bmj.com/site/about/guidelines.xhtml

To explore the extent to which there may be a differential treatment effect by presence of *Ureaplasma spp.*, the model fitted for the primary analysis will be extended by including a main and treatment group interaction term for *Ureaplasma* spp. colonisation at baseline. Since patterns of *Ureaplasma* colonisation may vary[22 32], the approach to this analysis may be modified in respect of emerging trends identified the placebo group samples. The final analytical approach will be agreed prior to database lock. Estimates from the statistical models (main effects and interaction terms) will be presented alongside 95% confidence intervals and p-values.

There are no formal stopping rules but the safety data will be regularly reviewed by the Trial Management Group, and at least annually by the independent Data Monitoring Committee.

Microbiology

Respiratory samples are being processed via centrifugation to separate the supernatant and obtain a cell pellet from which DNA will be extracted. Identification *of Ureaplasma urealyticum*, *Ureaplasma parvum* and *Mycoplasma hominis* is being performed using multiplex quantitative polymerase chain reaction (qPCR) assay as previously described [33 34]. All DNA extractions and qPCR assays are being performed in collaboration with Central Biotechnology Services (Cardiff University, UK) according to standard operating procedures and GCP to minimise contamination.

Stool samples are being cultured aerobically and non-aerobically and in the presence and absence of azithromycin to assess baseline and new development of azithromycin-resistant organisms.

Discussion

As the survival of preterm infants increases, decreasing morbidity associated with prematurity including CLD is becoming increasingly important. AZTEC is an example of evaluating drug development [35] in infants as a large, pragmatic, adequately powered trial of a macrolide antibiotic to prevent development of CLD with widespread support by the neonatal community [36 37]. The importance of improving outcomes for preterm infants at risk of developing CLD is highlighted by the potential impacts on later pulmonary function, especially the prospect of early decline in lung function and early-onset COPD [38]. Moreover, the evidence base to treat graduates of the neonatal unit when they present with symptoms in childhood is poor [39], largely because the mechanisms of prematurity-associated respiratory morbidity remain poorly understood [40].

Whether modification of pulmonary inflammation and treatment of *Ureaplasma* infection would reduce rates of CLD has been debated for three decades. Use of postnatal corticosteroids is associated with long term adverse effects, however the safety profile of azithromycin is superior and

For peer review only - http://bmjopen.bmj.com/site/about/guidelines.xhtml

is unlikely to affect neurological outcomes. Plans to follow up neurodevelopment and respiratory outcomes at one and two years of age are in place. This study includes ascertainment of whether each infant is colonised with *Ureaplasma* spp. and randomisation of treatment to azithromycin or placebo. Given the time lag between randomisation and the results of tests for *Ureaplasma* spp. the randomisation will not be balanced for *Ureaplasma* status. Nevertheless, this study design will allow an assessment of the causative role of *Ureaplasma* in the development of CLD.

If effectiveness of azithromycin therapy is demonstrated, integration into standard care would be relatively straightforward since the majority of candidates for treatment would have I.V. access for 7-10 days. Establishing whether azithromycin alters antibiotic resistance patterns will provide important information to the clinician who may have concerns around adding an adjunct treatment to a wide array of antimicrobials already given to this vulnerable group of patients. Although AZTEC will not specifically address mechanistic actions of azithromycin including its effect on the lung and gut microbiota, samples will be banked to permit such work in the near future using established [25], and novel methodologies such as metagenomics [41].

In summary, the AZTEC trial is addressing an important therapeutic need in an area where treatment based on high-quality evidence is severely lacking. Should antimicrobial resistance not be affected, azithromycin therapy should be appealing to clinicians due to the anti-inflammatory properties and proven ability to eradicate *Ureaplasma.* Treatment may yield fewer intensive care days and fewer discharges on home oxygen resulting in considerable reduction in costs to the NHS, as well as decreasing the significant burden on parents. On the other hand, should the treatment not be clinically effective the neonatal research community can turn their attention to alternative options for combatting CLD. Identification of an effective treatment to prevent CLD would be of exceptional value to the NHS, infants and their families

Ethics and dissemination

The current version of the AZTEC protocol is 3.0, dated 20/06/2020. Ethics permission has granted by the Wales Research Ethics Committee 2 (Ref 18/WA/0199), and regulatory permission by the Medicines and Healthcare Products Regulatory Agency (CTA reference 21323/0050/001-0001). NHS permission has been granted by the Health Research Authority and capacity and capability confirmed by each individual NHS organisation. The study is registered on EudraCT (2018-001109-99), ISRCTN (ISRCTN11650227) and on the National Institute for Health Research portfolio (CPMS 39385). Cardiff University is the Sponsor (resgov@cardiff.ac.uk), and were not involved in the preparation of this manuscript or the decision to submit. Personal data is held with the explicit consent of participants, independently of study data. The Centre for Trials Research has policies and

For peer review only - http://bmjopen.bmj.com/site/about/guidelines.xhtml

procedures relating to data requests: https://www.cardiff.ac.uk/centre-for-trials-research/about-us/data-requests. We shall disseminate our findings via national and international peer-reviewed journals, and conferences. A summary of the findings will also be posted on the trial website.

Oversight of the study is being performed by an independent Data Monitoring Committee (comprised of two expert neonatologists, and an expert statistician) and an independent Trial Steering Committee (comprised of two expert neonatologists, an expert statistician and a lay representative). Appointments to these committees were made with approval of the NIHR HRA and meeting a being held at least annually.

Patient and public involvement

Parental input was obtained during the grant application on the design and conduct of the study. Parent representatives also reviewed public-facing information (e.g. information sheets and consent form) and are members of the Trial Management Group. An independent parent representative is a member of the Trial Steering Committee.

Authors contributions

SK is the Chief Investigator who developed the research question and secured funding together with JL, MT, and JB with subsequent input from JRM, NK, KH, DG, and ETJ. JL and SK wrote the protocol with input from all authors. JL is the Trial Manager, managed by ETJ Senior Trial Manager, and is responsible for coordinating the operational delivery of the protocol and recruitment. DG and TP are the trial statisticians and authored the statistical analysis plan. JL and NKi led the development of the case report forms; NKi supervises data management. LZ is the laboratory analyst; expert microbiology advice is provided by NK and JRM. SK, MT, MH and JB provide expert clinical input; MH provides nursing and clinical expertise. JL wrote the first draft of the manuscript supervised by SK. All authors provided critical review and final approval of the manuscript.

Funding statement

This work is supported by the National Institute of Health Research Health Technology Assessment programme (Ref 16/111/106). The study duration is 01/01/2019-30/09/2022.

For peer review only - http://bmjopen.bmj.com/site/about/guidelines.xhtml

Competing interests statement

SK declares research grant funding from the NIHR, MRC and JP Moulton Charitable Foundation for the submitted work; SK also declares personal fees from Aspire Pharma Ltd. JL, DG, LZ, ETJ, MT, NK, JRM and JB declare funding from the NIHR for the submitted work. The other authors have no competing interests to declare.

Acknowledgements

We are very grateful for the support provided from both the independent Data Monitoring Committee and Trial Steering Committee. The Centre for Trials Research receives core funding from the Welsh Government via Health & Care Research Wales and Cancer Research UK. We would like to acknowledge and thank the expert help we received from many parents and nursing staff to develop the initial application and protocol. In particular, we would like to thank the babies and their parents who have started to be enrolled to AZTEC.

For peer review only

For peer review only - http://bmjopen.bmj.com/site/about/guidelines.xhtml

References

1. Simpson SJ, Turkovic L, Wilson AC, et al. Lung function trajectories throughout childhood in survivors of very preterm birth: a longitudinal cohort study. *Lancet Child Adolesc Health* 2018;2(5):350-59. doi: 10.1016/s2352-4642(18)30064-6 [published Online First: 2018/09/01]
2. Edwards MO, Kotecha SJ, Lowe J, et al. Management of Prematurity-Associated Wheeze and Its Association with Atopy. *PLoS One* 2016;11(5):e0155695. doi: 10.1371/journal.pone.0155695 [published Online First: 2016/05/21]
3. Kotecha SJ, Edwards MO, Watkins WJ, et al. Effect of preterm birth on later FEV1: a systematic review and meta-analysis. *Thorax* 2013;68(8):760-6. doi: 10.1136/thoraxjnl-2012-203079 [published Online First: 2013/04/23]
4. Blencowe H, Cousens S, Oestergaard MZ, et al. National, regional, and worldwide estimates of preterm birth rates in the year 2010 with time trends since 1990 for selected countries: a systematic analysis and implications. *Lancet* 2012;379(9832):2162-72. doi: 10.1016/s0140-6736(12)60820-4 [published Online First: 2012/06/12]
5. Higgins RD, Jobe AH, Koso-Thomas M, et al. Bronchopulmonary Dysplasia: Executive Summary of a Workshop. *J Pediatr* 2018;197:300-08. doi: 10.1016/j.jpeds.2018.01.043 [published Online First: 2018/03/20]
6. Kotecha S. Lung growth for beginners. *Paediatr Respir Rev* 2000;1(4):308-13. doi: 10.1053/prrv.2000.0069 [published Online First: 2005/11/03]
7. Chakraborty M, McGreal EP, Kotecha S. Acute lung injury in preterm newborn infants: mechanisms and management. *Paediatr Respir Rev* 2010;11(3):162-70; quiz 70. doi: 10.1016/j.prrv.2010.03.002 [published Online First: 2010/08/10]
8. Abman SH, Bancalari E, Jobe A. The Evolution of Bronchopulmonary Dysplasia after 50 Years. *Am J Respir Crit Care Med* 2017;195(4):421-24. doi: 10.1164/rccm.201611-2386ED [published Online First: 2017/02/16]
9. Kotecha S, Chan B, Azam N, et al. Increase in interleukin-8 and soluble intercellular adhesion molecule-1 in bronchoalveolar lavage fluid from premature infants who develop chronic lung disease. *Arch Dis Child Fetal Neonatal Ed* 1995;72(2):F90-6. doi: 10.1136/fn.72.2.f90 [published Online First: 1995/03/01]
10. Maxwell NC, Davies PL, Kotecha S. Antenatal infection and inflammation: what's new? *Curr Opin Infect Dis* 2006;19(3):253-8. doi: 10.1097/01.qco.0000224819.42729.2e [published Online First: 2006/04/29]
11. Waites KB, Schelonka RL, Xiao L, et al. Congenital and opportunistic infections: Ureaplasma species and Mycoplasma hominis. *Semin Fetal Neonatal Med* 2009;14(4):190-9. doi: 10.1016/j.siny.2008.11.009 [published Online First: 2008/12/26]
12. Lowe J, Watkins WJ, Edwards MO, et al. Association between pulmonary ureaplasma colonization and bronchopulmonary dysplasia in preterm infants: updated systematic review and meta-analysis. *Pediatr Infect Dis J* 2014;33(7):697-702. doi: 10.1097/INF.0000000000000239 [published Online First: 2014/01/22]
13. Filippone M, Nardo D, Bonadies L, et al. Update on Postnatal Corticosteroids to Prevent or Treat Bronchopulmonary Dysplasia. *Am J Perinatol* 2019;36(S 02):S58-s62. doi: 10.1055/s-0039-1691802 [published Online First: 2019/06/27]
14. Mabanta CG, Pryhuber GS, Weinberg GA, et al. Erythromycin for the prevention of chronic lung disease in intubated preterm infants at risk for, or colonized or infected with Ureaplasma urealyticum. *Cochrane Database Syst Rev* 2003(4):CD003744. doi: 10.1002/14651858.CD003744 [published Online First: 2003/10/30]
15. Ozdemir R, Erdeve O, Dizdar EA, et al. Clarithromycin in preventing bronchopulmonary dysplasia in Ureaplasma urealyticum-positive preterm infants. *Pediatrics* 2011;128(6):e1496-501. doi: 10.1542/peds.2011-1350 [published Online First: 2011/11/30]

For peer review only - http://bmjopen.bmj.com/site/about/guidelines.xhtml

16. Alvarez-Elcoro S, Enzler MJ. The macrolides: erythromycin, clarithromycin, and azithromycin. *Mayo Clin Proc* 1999;74(6):613-34. doi: 10.4065/74.6.613 [published Online First: 1999/06/23]
17. Aghai ZH, Kode A, Saslow JG, et al. Azithromycin suppresses activation of nuclear factor-kappa B and synthesis of pro-inflammatory cytokines in tracheal aspirate cells from premature infants. *Pediatr Res* 2007;62(4):483-8. doi: 10.1203/PDR.0b013e318142582d [published Online First: 2007/08/02]
18. Legrand T, Elie V, Kotecha S, et al. An optimal LC-MS/MS method for determination of azithromycin in white blood cells: application to pediatric samples. *Bioanalysis* 2014;6(17):2317-28. doi: 10.4155/bio.14.81 [published Online First: 2014/11/11]
19. Nair V, Loganathan P, Soraisham AS. Azithromycin and other macrolides for prevention of bronchopulmonary dysplasia: a systematic review and meta-analysis. *Neonatology* 2014;106(4):337-47. doi: 10.1159/000363493 [published Online First: 2014/10/04]
20. Hassan HE, Othman AA, Eddington ND, et al. Pharmacokinetics, safety, and biologic effects of azithromycin in extremely preterm infants at risk for ureaplasma colonization and bronchopulmonary dysplasia. *J Clin Pharmacol* 2011;51(9):1264-75. doi: 10.1177/0091270010382021 [published Online First: 2010/11/26]
21. Merchan LM, Hassan HE, Terrin ML, et al. Pharmacokinetics, microbial response, and pulmonary outcomes of multidose intravenous azithromycin in preterm infants at risk for Ureaplasma respiratory colonization. *Antimicrob Agents Chemother* 2015;59(1):570-8. doi: 10.1128/AAC.03951-14 [published Online First: 2014/11/12]
22. Viscardi RM, Terrin ML, Magder LS, et al. Randomised trial of azithromycin to eradicate Ureaplasma in preterm infants. *Archives of Disease in Childhood - Fetal and Neonatal Edition* 2020:fetalneonatal-2019-318122. doi: 10.1136/archdischild-2019-318122
23. Smith C, Egunsola O, Choonara I, et al. Use and safety of azithromycin in neonates: a systematic review. *BMJ Open* 2015;5(12):e008194. doi: 10.1136/bmjopen-2015-008194 [published Online First: 2015/12/15]
24. Beeton ML, Chalker VJ, Jones LC, et al. Antibiotic Resistance among Clinical Ureaplasma Isolates Recovered from Neonates in England and Wales between 2007 and 2013. *Antimicrob Agents Chemother* 2016;60(1):52-6. doi: 10.1128/AAC.00889-15 [published Online First: 2015/10/16]
25. Gallacher D, Mitchell E, Alber D, et al. Dissimilarity of the gut-lung axis and dysbiosis of the lower airways in ventilated preterm infants. *Eur Respir J* 2020 doi: 10.1183/13993003.01909-2019 [published Online First: 2020/02/16]
26. Bruynseels D, Solomon C, Hallam A, et al. Commentary on Reconstituting Fibrinogen Concentrate to Maintain Blinding in a Double-blind, Randomized Trial in an Emergency Setting. *J Emerg Med* 2016;50(1):104-7 e1. doi: 10.1016/j.jemermed.2015.09.017 [published Online First: 2015/10/29]
27. Jobe AH, Bancalari E. Bronchopulmonary dysplasia. *Am J Respir Crit Care Med* 2001;163(7):1723-9. doi: 10.1164/ajrccm.163.7.2011060 [published Online First: 2001/06/13]
28. Quine D, Wong CM, Boyle EM, et al. Non-invasive measurement of reduced ventilation:perfusion ratio and shunt in infants with bronchopulmonary dysplasia: a physiological definition of the disease. *Arch Dis Child Fetal Neonatal Ed* 2006;91(6):F409-14. doi: 10.1136/adc.2006.095406 [published Online First: 2006/06/27]
29. The BOOST-II Australia and United Kingdom Collaborative Groups. Outcomes of Two Trials of Oxygen-Saturation Targets in Preterm Infants. *New England Journal of Medicine* 2016;374(8):749-60. doi: 10.1056/NEJMoa1514212
30. Moher D, Schulz KF, Altman D. The CONSORT statement: revised recommendations for improving the quality of reports of parallel-group randomized trials. *Jama* 2001;285(15):1987-91. doi: 10.1001/jama.285.15.1987 [published Online First: 2001/04/20]

For peer review only - http://bmjopen.bmj.com/site/about/guidelines.xhtml

31. International Council for Harmonisation. ICH E9: Statistical Principles for Clinical Trials, Step 5 1998 [Available from: https://www.ema.europa.eu/en/documents/scientific-guideline/ich-e-9-statistical-principles-clinical-trials-step-5_en.pdf accessed 17/04/2020 2020.
32. Castro-Alcaraz S, Greenberg EM, Bateman DA, et al. Patterns of colonization with Ureaplasma urealyticum during neonatal intensive care unit hospitalizations of very low birth weight infants and the development of chronic lung disease. *Pediatrics* 2002;110(4):e45. doi: 10.1542/peds.110.4.e45 [published Online First: 2002/10/03]
33. Payne MS, Goss KC, Connett GJ, et al. A quantitative analysis of Ureaplasma urealyticum and Ureaplasma parvum compared with host immune response in preterm neonates at risk of developing bronchopulmonary dysplasia. *J Clin Microbiol* 2012;50(3):909-14. doi: 10.1128/JCM.06625-11 [published Online First: 2011/12/23]
34. Férandon C, Peuchant O, Janis C, et al. Development of a real-time PCR targeting the yidC gene for the detection of Mycoplasma hominis and comparison with quantitative culture. *Clin Microbiol Infect* 2011;17(2):155-9. doi: 10.1111/j.1469-0691.2010.03217.x [published Online First: 2010/03/20]
35. Turner MA, Jacqz-Aigrain E, Kotecha S. Azithromycin, Ureaplasma and chronic lung disease of prematurity: a case study for neonatal drug development. *Arch Dis Child* 2012;97(6):573-7. doi: 10.1136/adc.2010.195180 [published Online First: 2011/06/24]
36. Maxwell NC, Nuttall D, Kotecha S. Does Ureaplasma spp. cause chronic lung disease of prematurity: ask the audience? *Early Hum Dev* 2009;85(5):291-6. doi: 10.1016/j.earlhumdev.2008.12.002 [published Online First: 2009/01/16]
37. Pansieri C, Pandolfini C, Elie V, et al. Ureaplasma, bronchopulmonary dysplasia, and azithromycin in European neonatal intensive care units: a survey. *Sci Rep* 2014;4:4076. doi: 10.1038/srep04076 [published Online First: 2014/02/13]
38. Stocks J, Hislop A, Sonnappa S. Early lung development: lifelong effect on respiratory health and disease. *Lancet Respir Med* 2013;1(9):728-42. doi: 10.1016/s2213-2600(13)70118-8 [published Online First: 2014/01/17]
39. Duijts L, van Meel ER, Moschino L, et al. European Respiratory Society guideline on long-term management of children with bronchopulmonary dysplasia. *Eur Respir J* 2020;55(1) doi: 10.1183/13993003.00788-2019 [published Online First: 2019/09/29]
40. Lowe J, Kotecha SJ, Kotecha S. Long Term Effects Following Extreme Prematurity: Respiratory Problems. In: Boyle EM, Cusack J, eds. Emerging Topics and Controversies in Neonatology. Switzerland: Springer 2020:351-66.
41. Mack I, Sharland M, Berkley JA, et al. Antimicrobial Resistance Following Azithromycin Mass Drug Administration: Potential Surveillance Strategies to Assess Public Health Impact. *Clin Infect Dis* 2020;70(7):1501-08. doi: 10.1093/cid/ciz893 [published Online First: 2019/10/22]

For peer review only

Captions for figures

Figure 1: Flow diagram for assessment of CLD severity in the AZTEC trial. Modified from the original https://www.npeu.ox.ac.uk/downloads/files/baby-oscar/protocol/Baby-OSCAR%20Protocol%20v6_171116.pdf

Figure 2: Flow diagram for AZTEC study procedures and follow-up. ETA= endotracheal aspirates, NPA= nasopharyngeal aspirates

Figure 3: AZTEC biological sampling plan Day 4-6 is acceptable for the day 5 samples. Day 8-12 is acceptable for the day 10 sample. Day 14-21- one sample of each type during this period.*

For peer review only - http://bmjopen.bmj.com/site/about/guidelines.xhtml

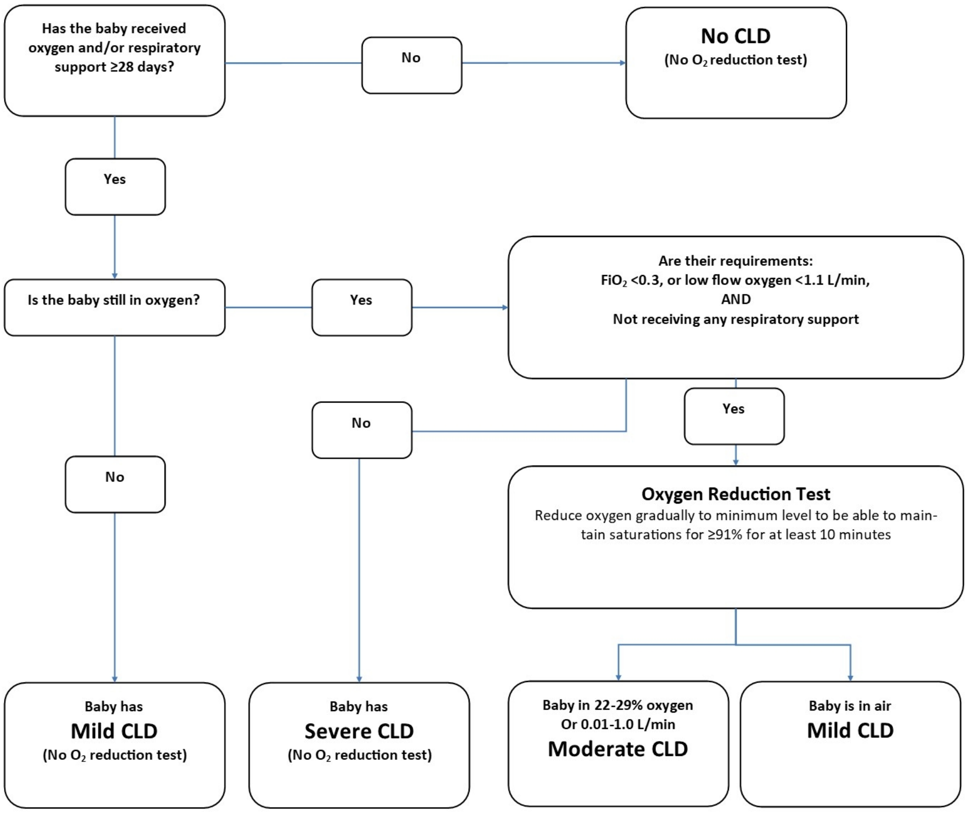

Figure 1: Flow diagram for assessment of CLD severity in the AZTEC trial. Modified from the original https://www.npeu.ox.ac.uk/downloads/files/baby-oscar/protocol/Baby-OSCAR%20Protocol%20v6_171116.pdf

178x149mm (200 x 200 DPI)

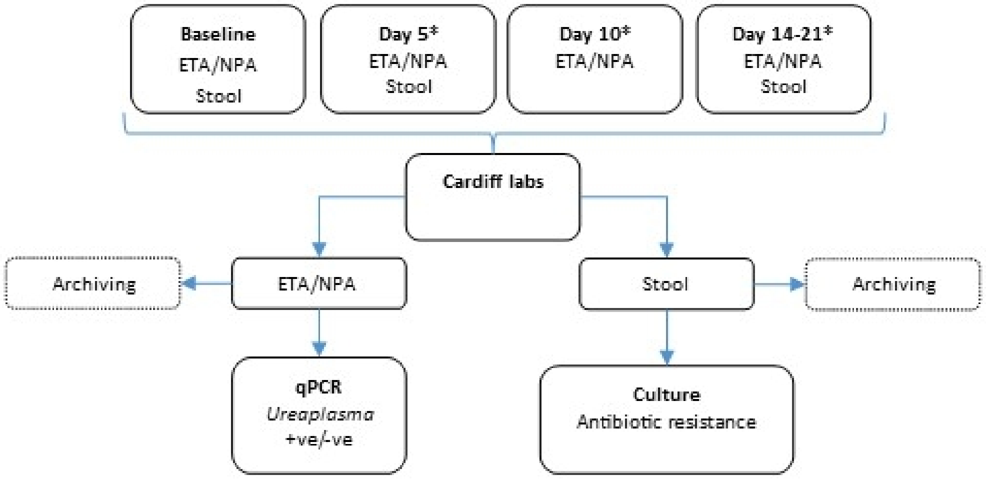

Figure 2: Flow diagram for AZTEC study procedures and follow-up. ETA= endotracheal aspirates, NPA= nasopharyngeal aspirates

130x63mm (100 x 100 DPI)

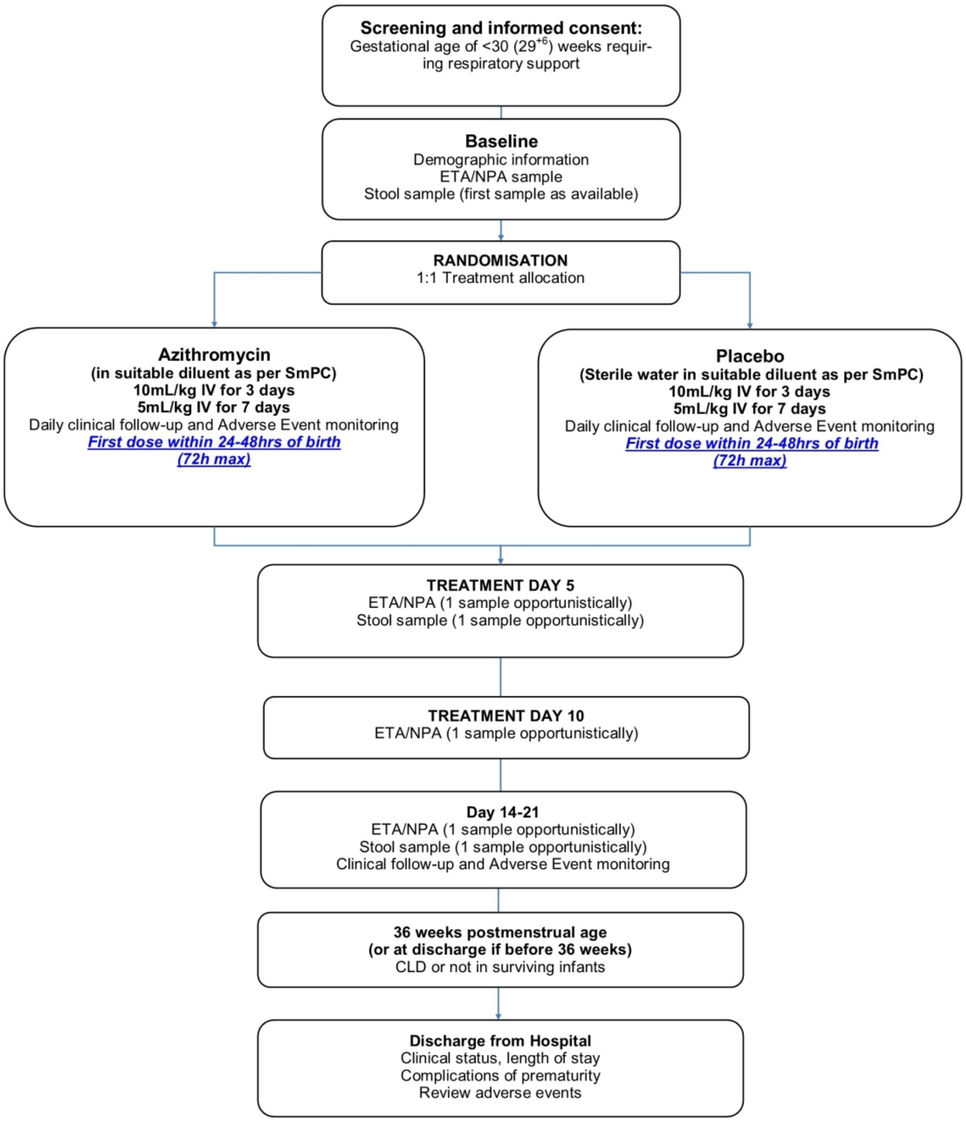

Figure 3: AZTEC biological sampling plan* Day 4-6 is acceptable for the day 5 samples. Day 8-12 is acceptable for the day 10 sample. Day 14-21- one sample of each type during this period.

175x214mm (300 x 300 DPI)

Centre for Trials Research
Canolfan Ymchwil Treialon
The Newcastle upon Tyne Hospitals NHS Foundation Trust
FUNDED BY NIHR National Institute for Health Research

<<NHS Organisation Logo>>
<Trust/Site address 1>
<Trust/Site address 2>
<Trust/Site address 3>
<postcode>

AZTEC

Azithromycin Therapy for Chronic Lung Disease

Parent Information Sheet

Summary

This is a brief summary of a research study called **AZTEC**. We would like to invite your baby to join our study because your baby may be, or has been, born early.

Babies born early are at risk of developing lung problems. Giving an extra medicine called azithromycin, (an ‘antibiotic’), to a baby born early, may be able help their lung problems. However, at the moment, we do not know whether babies who have this medicine will benefit compared to babies who do not receive the medicine. Our hospital is one of many helping in a UK-wide study to find out whether the medicine should be used routinely in the future for premature babies. We would like approximately 800 families to take part, in total.

If, after reading this information and talking with others, you wish for your baby to take part, we will ask you to sign a consent form and your baby will then be entered into the study. We will collect information about your baby’s progress in hospital and would like to follow them up at one and two years of age.

Taking part in the study is entirely up to you. If you decide not to take part, this will not affect the care your baby receives. You do not have to give a reason if you do not want to take part, although providing your reasons may be helpful to us if you are willing. The rest of this leaflet explains the study in more detail and describes what being in the study would mean for you and your baby.

Thank you for taking the time to read this leaflet.

We would like to invite your baby to take part in our study called AZTEC

We understand and appreciate that this is a very difficult time for you and your family. However, we think it is important that you know about a study that this hospital is taking part in for babies born early.

We would like to ask your permission for your baby to take part in this study. Before you decide, it is important for you to understand why the research is being done and what is involved.

What is the AZTEC Study about?

Babies born early (under 30 weeks) are at risk of developing lung problems after birth. A major reason for this is that the lungs are not fully developed. This often means that after birth, babies born early may need some extra medicines and sometimes help with their breathing. Babies born early often develop inflammation (soreness and redness) of their lungs, sometimes because of germs (including one called *Ureaplasma)*.

Azithromycin is a medicine which can reduce inflammation and treats infection in the lungs. Reducing either, or both of these might help the lung problems of babies born early. The best way to find out is to compare babies who are given azithromycin with those who are not.

Why has my baby been asked to take part?

We are inviting your baby to take part in this study because they are a patient at one of the hospitals taking part and have been born under 30 weeks of pregnancy and need some help with their breathing.

Does my baby have to take part?

No, taking part is voluntary. It is up to you to decide whether or not you want your baby to take part. If you choose to let your baby take part you can also choose to stop at any time without giving a reason, although providing your reasons may be helpful to us if you are

For peer review only - http://bmjopen.bmj.com/site/about/guidelines.xhtml

willing. The standard of care your baby will receive now or in the future will be the same whether they take part or not.

What will my baby and I have to do if we take part?

A member of the clinical team will talk to you first in more detail and you will be able to ask any questions that you have. If you have had all of your questions answered and are happy for your baby to take part then you will be asked to sign a consent form to confirm you would like your baby to participate. You will be given a copy of the consent form and the information sheet to keep.

In research studies, we often test the medicine against a dummy medicine (also called a placebo) to see if the medicine works. A computer program will decide if your baby receives the azithromycin once a day for ten days or the dummy medicine, (water), once a day for ten days. Neither you, nor the team looking after your baby will know which medicine the baby is on. However, if the team looking after your baby need to know what your baby is receiving then they can find out quickly. The germs may be acquired before birth or at delivery, so it is best to start the treatment as early as possible and within 72 hours of birth.

We will also collect fluid samples from your baby's breathing tubes or from their nose. This is done regularly for all babies who need help with their breathing, but the fluid is not normally kept. The fluid will be tested for germs and inflammation. Samples of your baby's stool (poo) will also be taken to look if azithromycin has an effect on normal bacteria. All of these will only be done if your baby is well enough. All other aspects of your baby's care will be exactly the same as if they were not in the study.

We shall collect details of your baby until 36 weeks of gestation or until the baby leaves the hospital if this is sooner. Some babies need oxygen for a variable length of time to help with their breathing - if your baby needs oxygen at 36 weeks' gestation then we shall carry out a short test to find out how much oxygen they require.

To find out if the treatment improves breathing when your baby grows up, we would like to contact you and your GP when your baby is one and two years old to see how they are doing. We will ask you to fill in questionnaires about their breathing and general health. We would also like to use routinely collected health data (information hospitals collect when looking after your baby), which is held by this hospital, NHS Digital, and from the National Neonatal Research database, which collects data on the follow-up of premature babies and children. Information such as your baby's name, NHS/CHI number will be used for this purpose, but the data viewed by Cardiff University researchers will not be identifiable. All information will be stored securely.

Timing of treatment and sample collection

Before study treatment → **Before study treatment**
The research team will explain the study to you and offer your baby the opportunity to take part. A lung fluid sample will be taken and stool (poo) sample will be taken (when available)

Day 1 → **Day 1- 10**
Study medicine or placebo (dummy) given once daily

Day 5 → **Day 5**
A fluid sample will be taken and a stool (poo) sample will be taken (when available)

Day 10 → **Day 10**
A fluid sample will be taken (when available)

Day 11-13 → **Day 11-13**
Regular follow-up and monitoring

Day 14 (up to 21 days) → **Day 14**
A fluid sample will be taken and a stool (poo) sample will be taken (when available)

36 weeks' gestation (prior to discharge if sooner) → **36 weeks post menstrual age**
Assess baby's requirement for oxygen

1 and 2 years corrected age → **1 and 2 years corrected age**
Questionnaires to be completed; access to healthcare databases obtained

For peer review only - http://bmjopen.bmj.com/site/about/guidelines.xhtml

What are the benefits and risks of my baby taking part?

Azithromycin has been used for a long time in children and has been used in other research studies in premature babies. Like all medicines, antibiotics can sometimes cause side-effects; these are uncommon, but we know that some babies may develop some soreness of the tummy or slightly looser stools. In older patients, azithromycin may affect the rhythm of the heart. There is no evidence that this happens in babies, but it is something we will monitor closely.

Although there are no certain benefits to you or your baby in taking part in the study, we hope the information we get might improve treatment and health outcomes for other babies in the future.

Will my baby's participation be kept confidential?

Yes. All the information collected about your baby during the study will be handled according to all applicable ethical and legal requirements.

Your baby's personal information will be kept strictly confidential and will only be accessed by people working on the study or working to ensure the study is being run correctly.

Your baby will be allocated a study number, which will be used to identify them on each paper form. Your full name, your baby's full name, and your baby's NHS number will be included on their consent form and a copy of this will be sent to the coordinating centre for the study, the Centre for Trials Research (CTR), based at Cardiff University. Every effort will be made to ensure that any information about your baby that leaves the hospital will have your baby's identifiers removed so that your baby cannot be recognised from it; this information will usually be removed by a member of the study team at your baby's hospital but may also be removed by the CTR upon receipt.

What will happen to the samples from my baby?

Any samples taken will be stored securely until they are transferred to Cardiff University. We shall test the samples in our laboratories at Cardiff, but some tests may be done by other university laboratories or commercial companies who have the appropriate expertise. In all cases, samples will be given a special code so that the person doing the laboratory tests will not know which baby the sample came from. We will record results on a special database where all the information is linked to this code. We shall use the baby's nose, lung and stool samples to look for DNA from germs. When we extract the DNA from the samples, DNA from both the baby and germs is unavoidably extracted. For this study we shall not use the baby's DNA. However, with your permission any remaining samples, including nose and lung fluid, stool, and DNA (baby and bacterial), may be stored for future research into chronic lung disease of prematurity.

The samples will be anonymised before use in future studies and may be accessed by researchers in the UK and abroad. The research may include genetic (e.g. DNA) or commercial research. You may withdraw your consent for the storage and future use of your baby's samples at any point by contacting the Centre for Trials Research (www.cardiff.ac.uk/centre-for-trials-research).

If you do withdraw your consent your baby's samples will not be used in any subsequent studies and will be destroyed according to locally approved practices. Any samples already distributed for use in research prior to the withdrawal of consent will continue to be used in that study and any samples remaining at the end of the study will be destroyed.

What happens if I change my mind about my baby taking part?

If at any point you decide you want your baby to stop taking part in the study, the care your baby receives will not be affected. If you do decide for your baby to stop taking part we would stop giving study treatment (if not finished) and ask you if you would like your baby to:

- continue to complete study follow up (data collection and whether stool/fluid samples can be taken)
- continue to be followed up at 1 and at 2 years of age, or
- stop taking part with no more study follow up.

If you withdraw your baby from the study, we will keep the information about your baby that we have already obtained. We may be required to collect some limited information about your baby and about side effects your baby may have as a result of taking part in the trial. This will only be collected if required by the Regulatory Authorities.

For peer review only - http://bmjopen.bmj.com/site/about/guidelines.xhtml

What will happen to the results of the study?

We aim to publish the results in medical journals. Confidentiality will be ensured at all times and you or your baby will not be identified in any publications. A summary will be published on the study website.

What if there is a problem?

If you have a concern about any aspect of this study, you should ask to speak with one of your baby's research team who will do their best to answer your questions. If you remain unhappy and wish to complain formally, you can do this through the NHS Complaints Procedure. Details can be obtained from the hospital.

If in the unlikely event your baby has been harmed by taking part in this study, you may have grounds for legal action and could seek compensation through the sponsor, who has appropriate insurance-related arrangements in place. If your baby is harmed and it is due to any routine clinical treatment or negligence, then the <<hospital institution>> indemnity arrangements will apply. If you wish to complain about any aspect of the way you or your baby has been treated, you may use the normal <<hospital institution>> complaints procedures; <<hospital advice service>> at your hospital will advise you about this (their contact details are at the end of this leaflet).

Additional information

Cardiff University is the sponsor and the data controller for this study. This means that they are responsible for looking after your baby's information and using it properly. They will be using information from your baby's medical records in order to undertake this study. The CTR at Cardiff University will keep identifiable information about your baby for 25 years after the study has finished. Arrangements for confidential destruction will then be made.

Your rights to access, change or move your baby's information are limited, as your baby's information needs to be managed in specific ways in order for the research to be reliable and accurate. If you withdraw your baby from the study, Cardiff University will keep the information about your baby that we have already obtained. To safeguard you and your baby's rights, they will use the minimum personally-identifiable information possible.

This hospital will use your baby's name, NHS/CHI number and contact details to make sure that relevant information about the study is recorded for your baby's care, and to oversee the quality of the study. This hospital will pass these details to Cardiff University so individuals from Cardiff University, and regulatory organisations may look at your baby's medical and research records to check the accuracy of the research study. The only other people at Cardiff who will have access to information that identifies your baby will be people who need to contact you for study follow-up or to audit the data collection process. This hospital will keep identifiable information about you from this study for 25 years after the study has finished.

AZTEC is funded by National Institute of Health Research, HTA Programme (HTA Project: 16/111/106). The study is registered on a public database of clinical trials called ISRCTN (ISRCTN11650227). For further details of national databases reviewed for this study please refer to the website <<URL>>. You can also find out more about how your information will be used on these webpages.

The research has been approved by Wales Research Ethics Committee 2, NHS Heath Research Authority, and by the Medicines and Healthcare Products Regulatory Agency (MHRA) who have agreed this study is being conducted in an appropriate manner.

Contacts for further information

If you would like more information or have any questions about the AZTEC study, please talk to:

Principal Investigator: **<PI NAME>**

Research Nurse: **<RN NAME>**

Telephone: **<number>**

Or visit the website: <<URL>>

If you wish to discuss the study with someone independent of the research team you can contact the local NHS Patient Advice and Liaison Service (PALS) on: <telephone number>

Thank you taking the time to read this information sheet

AZTEC
Azithromycin Therapy for Chronic Lung Disease

<<NHS Organisation Logo>>
<Trust/Site address 1>
<Trust/Site address 2>
<Trust/Site address 3>
<postcode>

Consent Form

Study ID: A Z _ _ _ _ ***NHS/CHI NUMBER:*** _ _ _ _ _ _ _ _ _ _

To be completed by the Parent/Guardian

Once you have read and understood each statement please enter your initials in each box	Initial
1. I have read and understood the information sheet for the AZTEC trial (Version 5.0, dated 20/06/2019). I have had the opportunity to ask questions and have had these answered satisfactorily.	initial
2. I understand that my baby's participation is voluntary and that I am free to withdraw my baby from the study at any time, without giving a reason, and without my baby's care or legal rights being affected. I understand that in some cases further information about any unwanted effects of my baby's treatment may need to be collected by the study team.	initial
3. I understand that my baby's data will be retained for a maximum of 25 years and that they will be stored in a confidential manner.	initial
4. I understand that information about both mother and baby that is held and maintained by NHS Digital and other central UK NHS bodies, may be collected from medical records and other health-related records. These may be looked at by the research team and responsible practitioners during the trial. The sponsor, NHS Organisation and Regulatory Authorities may also look at the data collected during the study. I give permission for these individuals to have access to these records and for them to be used in this research on the understanding that all information will remain confidential.	initial
5. I understand that samples of my baby's lung fluid, stool and nasal fluid will be collected for this study. I understand that my baby's DNA will be extracted alongside the bacterial DNA, but will not be used in the AZTEC study.	initial
6. I agree to allow information or results arising from this study to be used in future healthcare and/or medical research in an anonymised form.	initial
7. I give permission for a copy of my baby's consent form to be sent to the CTR at Cardiff University (where it will be kept in a secure location), to allow confirmation that my consent was given.	initial
8. I agree for my baby to take part in the above study.	initial
Below are optional statements:	
9. I agree for my baby's follow up data on applicable databases (at this hospital, NHS digital/NHS Wales Informatics Service/ISD Scotland and the National Neonatal Research Database) to be reviewed by Cardiff University researchers.	initial
10. I agree for me and my GP to be contacted by Cardiff University for the purposes of follow up when my baby is one and two years corrected age. I understand that contact details will be kept strictly confidential (according to the 2018 General Data Protection Regulation) and that no personal information will be used in study reports or publications.	initial
11. I agree to gift any of my baby's remaining samples (including my baby's DNA) to be used in future chronic lung disease of prematurity research in the UK and abroad, which may include genetic (e.g. DNA) and commercial research. I understand I am free to withdraw my consent to future research at any point and that all samples will be destroyed as detailed in the information sheet	initial
12. I agree that I may be contacted in the future in relation to other research studies	initial

Baby's name (please print): ______________________________

Name of parent/guardian (please print): ______________________________ Your signature: __________________________

Relationship to baby: ____________________________ Date: ______________

2nd Parent name, (please print): ____________________________ Your signature: __________________________ Date: ______________

Researcher name (please print): _____________________________ Signature: ______________________________ Date: ______________

For peer review only - http://bmjopen.bmj.com/site/about/guidelines.xhtml

STANDARD PROTOCOL ITEMS: RECOMMENDATIONS FOR INTERVENTIONAL TRIALS

SPIRIT 2013 Checklist: Recommended items to address in a clinical trial protocol and related documents*

Section/item	Item No	Description	Addressed on page number
**Administrative information**			
Title	1	Descriptive title identifying the study design, population, interventions, and, if applicable, trial acronym	1
Trial registration	2a	Trial identifier and registry name. If not yet registered, name of intended registry	2
	2b	All items from the World Health Organization Trial Registration Data Set	Throughout
Protocol version	3	Date and version identifier	15
Funding	4	Sources and types of financial, material, and other support	15
Roles and responsibilities	5a	Names, affiliations, and roles of protocol contributors	1, 16
	5b	Name and contact information for the trial sponsor	15
	5c	Role of study sponsor and funders, if any, in study design; collection, management, analysis, and interpretation of data; writing of the report; and the decision to submit the report for publication, including whether they will have ultimate authority over any of these activities	15
	5d	Composition, roles, and responsibilities of the coordinating centre, steering committee, endpoint adjudication committee, data management team, and other individuals or groups overseeing the trial, if applicable (see Item 21a for data monitoring committee)	15, 16

For peer review only

Introduction

Background and rationale	6a	Description of research question and justification for undertaking the trial, including summary of relevant studies (published and unpublished) examining benefits and harms for each intervention	3-4
	6b	Explanation for choice of comparators	3-4
Objectives	7	Specific objectives or hypotheses	5
Trial design	8	Description of trial design including type of trial (eg, parallel group, crossover, factorial, single group), allocation ratio, and framework (eg, superiority, equivalence, noninferiority, exploratory)	5

Methods: Participants, interventions, and outcomes

Study setting	9	Description of study settings (eg, community clinic, academic hospital) and list of countries where data will be collected. Reference to where list of study sites can be obtained	5
Eligibility criteria	10	Inclusion and exclusion criteria for participants. If applicable, eligibility criteria for study centres and individuals who will perform the interventions (eg, surgeons, psychotherapists)	5-6
Interventions	11a	Interventions for each group with sufficient detail to allow replication, including how and when they will be administered	6-7
	11b	Criteria for discontinuing or modifying allocated interventions for a given trial participant (eg, drug dose change in response to harms, participant request, or improving/worsening disease)	N/A
	11c	Strategies to improve adherence to intervention protocols, and any procedures for monitoring adherence (eg, drug tablet return, laboratory tests)	9
	11d	Relevant concomitant care and interventions that are permitted or prohibited during the trial	N/A
Outcomes	12	Primary, secondary, and other outcomes, including the specific measurement variable (eg, systolic blood pressure), analysis metric (eg, change from baseline, final value, time to event), method of aggregation (eg, median, proportion), and time point for each outcome. Explanation of the clinical relevance of chosen efficacy and harm outcomes is strongly recommended	7-9
Participant timeline	13	Time schedule of enrolment, interventions (including any run-ins and washouts), assessments, and visits for participants. A schematic diagram is highly recommended (see Figure)	10

For peer review only - http://bmjopen.bmj.com/site/about/guidelines.xhtml

Sample size	14	Estimated number of participants needed to achieve study objectives and how it was determined, including clinical and statistical assumptions supporting any sample size calculations	12-13
Recruitment	15	Strategies for achieving adequate participant enrolment to reach target sample size	9

Methods: Assignment of interventions (for controlled trials)

Allocation:

Sequence generation	16a	Method of generating the allocation sequence (eg, computer-generated random numbers), and list of any factors for stratification. To reduce predictability of a random sequence, details of any planned restriction (eg, blocking) should be provided in a separate document that is unavailable to those who enrol participants or assign interventions	11
Allocation concealment mechanism	16b	Mechanism of implementing the allocation sequence (eg, central telephone; sequentially numbered, opaque, sealed envelopes), describing any steps to conceal the sequence until interventions are assigned	11
Implementation	16c	Who will generate the allocation sequence, who will enrol participants, and who will assign participants to interventions	11
Blinding (masking)	17a	Who will be blinded after assignment to interventions (eg, trial participants, care providers, outcome assessors, data analysts), and how	11
	17b	If blinded, circumstances under which unblinding is permissible, and procedure for revealing a participant's allocated intervention during the trial	11

Methods: Data collection, management, and analysis

Data collection methods	18a	Plans for assessment and collection of outcome, baseline, and other trial data, including any related processes to promote data quality (eg, duplicate measurements, training of assessors) and a description of study instruments (eg, questionnaires, laboratory tests) along with their reliability and validity, if known. Reference to where data collection forms can be found, if not in the protocol	11-12
	18b	Plans to promote participant retention and complete follow-up, including list of any outcome data to be collected for participants who discontinue or deviate from intervention protocols	11-12

Data management	19	Plans for data entry, coding, security, and storage, including any related processes to promote data quality (eg, double data entry; range checks for data values). Reference to where details of data management procedures can be found, if not in the protocol	11-12
Statistical methods	20a	Statistical methods for analysing primary and secondary outcomes. Reference to where other details of the statistical analysis plan can be found, if not in the protocol	13
	20b	Methods for any additional analyses (eg, subgroup and adjusted analyses)	13
	20c	Definition of analysis population relating to protocol non-adherence (eg, as randomised analysis), and any statistical methods to handle missing data (eg, multiple imputation)	13
**Methods: Monitoring**			
Data monitoring	21a	Composition of data monitoring committee (DMC); summary of its role and reporting structure; statement of whether it is independent from the sponsor and competing interests; and reference to where further details about its charter can be found, if not in the protocol. Alternatively, an explanation of why a DMC is not needed	15
	21b	Description of any interim analyses and stopping guidelines, including who will have access to these interim results and make the final decision to terminate the trial	13
Harms	22	Plans for collecting, assessing, reporting, and managing solicited and spontaneously reported adverse events and other unintended effects of trial interventions or trial conduct	11
Auditing	23	Frequency and procedures for auditing trial conduct, if any, and whether the process will be independent from investigators and the sponsor	15
**Ethics and dissemination**			
Research ethics approval	24	Plans for seeking research ethics committee/institutional review board (REC/IRB) approval	15
Protocol amendments	25	Plans for communicating important protocol modifications (eg, changes to eligibility criteria, outcomes, analyses) to relevant parties (eg, investigators, REC/IRBs, trial participants, trial registries, journals, regulators)	15

Consent or assent	26a	Who will obtain informed consent or assent from potential trial participants or authorised surrogates, and how (see Item 32)	11
	26b	Additional consent provisions for collection and use of participant data and biological specimens in ancillary studies, if applicable	11
Confidentiality	27	How personal information about potential and enrolled participants will be collected, shared, and maintained in order to protect confidentiality before, during, and after the trial	15
Declaration of interests	28	Financial and other competing interests for principal investigators for the overall trial and each study site	16
Access to data	29	Statement of who will have access to the final trial dataset, and disclosure of contractual agreements that limit such access for investigators	15
Ancillary and post-trial care	30	Provisions, if any, for ancillary and post-trial care, and for compensation to those who suffer harm from trial participation	NA
Dissemination policy	31a	Plans for investigators and sponsor to communicate trial results to participants, healthcare professionals, the public, and other relevant groups (eg, via publication, reporting in results databases, or other data sharing arrangements), including any publication restrictions	15
	31b	Authorship eligibility guidelines and any intended use of professional writers	NA
	31c	Plans, if any, for granting public access to the full protocol, participant-level dataset, and statistical code	15
**Appendices**			
Informed consent materials	32	Model consent form and other related documentation given to participants and authorised surrogates	NA
Biological specimens	33	Plans for collection, laboratory evaluation, and storage of biological specimens for genetic or molecular analysis in the current trial and for future use in ancillary studies, if applicable	12

*It is strongly recommended that this checklist be read in conjunction with the SPIRIT 2013 Explanation & Elaboration for important clarification on the items. Amendments to the protocol should be tracked and dated. The SPIRIT checklist is copyrighted by the SPIRIT Group under the Creative Commons "Attribution-NonCommercial-NoDerivs 3.0 Unported" license.
